# Transcription Dependent Loss of an Ectopically Expressed Variant Surface Glycoprotein during Antigenic Variation in Trypanosoma brucei

**DOI:** 10.1128/mbio.03847-21

**Published:** 2022-03-01

**Authors:** Emilia Jane McLaughlin, Karinna Rubio-Pena, Annick Dujeancourt-Henry, Lucy Glover

**Affiliations:** a Institut Pasteur, Université de Paris, Trypanosome Molecular Biology, Department of Parasites and Insect Vectors, Paris, France; b Université de Paris, Sorbonne Paris Cité, Paris, France; University of Georgia

**Keywords:** *Trypanosoma brucei*, VSG, antigenic variation

## Abstract

In the mammalian host, Trypanosoma brucei is coated in a single-variant surface glycoprotein (VSG) species. Stochastic switching of the expressed *VSG* allows the parasite to escape detection by the host immune system. DNA double-strand breaks (DSB) trigger VSG switching, and repair via gene conversion results in an antigenically distinct *VSG* being expressed from the single active bloodstream-form expression site (BES). The single active BES is marked by *VSG* exclusion 2 (VEX2) protein. Here, we have disrupted monoallelic *VSG* expression by stably expressing a second telomeric *VSG* from a ribosomal locus. We found that cells expressing two *VSGs* contained one VEX2 focus that was significantly larger in size than the wild-type cells; this therefore suggests the ectopic *VSG* is expressed from the same nuclear position as the active BES. Unexpectedly, we report that in the double *VSG*-expressing cells, the DNA sequence of the ectopic copy is lost following a DSB in the active BES, despite it being spatially separated in the genome. The loss of the ectopic *VSG* is dependent on active transcription and does not disrupt the number or variety of templates used to repair a BES DSB and elicit a VSG switch. We propose that there are stringent mechanisms within the cell to reinforce monoallelic expression during antigenic variation.

## INTRODUCTION

Trypanosoma brucei resides in the extracellular spaces of its mammalian host (1, 2), where survival is dependent on the stochastic switching of the dense variant surface glycoprotein (VSG) coat, consisting of over 10 million molecules of a single VSG species (3). Trypanosomes have dedicated a remarkably large proportion of their genome to antigenic variation, with a repertoire of over 2,000 *VSG* genes, pseudogenes, and gene fragments found in the subtelomeric regions (4–6); however, only one *VSG* is expressed at any one time from a specialized subtelomeric locus known as a bloodstream-form expression site (BES). There are approximately 15 to 20 BESs in the T. brucei genome, and the structure is broadly conserved (7), with the *VSG* gene flanked upstream by a block of repetitive sequences known as the 70-bp repeats and downstream by the telomere (4, 5, 8). Unusually the single active *VSG* is transcribed by RNA polymerase I (RNA Pol I) (9, 10), which is normally reserved for the transcription of rRNA genes (11) at a specialized transcription compartment known as the expression site body (ESB) (9, 12, 13). The active BES occupies a distinct chromatin architecture, which is depleted of nucleosomes (14, 15) and is enriched for highly sumoylated chromatin-associated proteins (16) and the high-mobility group box (HMGB) chromatin protein TDP1 (17, 18). The recently identified VSG exclusion (VEX) complex, consisting of VEX1 and VEX2, localizes to the active BES in a transcription-dependent manner and defines the single active BES (19, 20). The chromatin assembly factor, CAF-1, maintains the epigenetic status of the active BES during DNA replication, and VEX complex association with the spliced leader locus ensures sufficient processing of the *VSG* mRNA (19–21).

To escape detection by the host immune system, the single expressed *VSG* is stochastically switched. *VSG* gene switching can occur by an *in situ* transcriptional BES switch (22, 23); however, the main pathway used is gene conversion (GC), which allows access to the subtelomeric archival *VSGs* via recombination (24). Antigenic variation is thought to be driven by DNA double-stranded breaks (DSB) that naturally accumulate in the BESs (25, 26), with the 70-bp repeats critical for recombination (5, 8, 27, 28). Indeed, disruption of factors involved in preserving genome integrity leads to elevated VSG switch frequency. Depletion of the RNases H1 and H2, which resolve RNA-DNA hybrids known as R-loops, increases replication-associated DNA damage and *VSG* switching (29, 30). RNase H2 interacts with the histone methyl transferase DOT1b, and *DOT1b* deletion results in RNA-DNA hybrids forming, DNA damage, and increased recombination-driven *VSG* switching (31). Similarly, knockdown of ataxia telangiectasia and Rad3-related (ATR) kinase increases recombination-driven VSG switching, which is associated with elevated levels of DNA damage (32, 33). The RTR complex (RECQ2-TOPO3a-RMI1) removes recombination intermediates during mitotic crossover, and loss of the *RECQ2* helicase or RMI1 deficiency leads to increased *VSG* gene switching by recombination (34, 35), while *TOPO3a* knockdown results in a RAD51-dependent hyperswitching phenotype (36). The telomere (37, 38), telomeric chromatin (39), nuclear lamina protein NUP-1 (40), and histone variants H3.V and H4.V (6) have also been shown to influence VSG switching.

While *VSG* expression is strictly monoallelic, this has been disrupted in the laboratory through the selection for activation of a second BES (41, 42) or by introducing a second *VSG* into the active BES (43–46) or an rDNA locus (41, 47, 48). While cell growth is not disrupted in parasites with a dual coat (42–44, 46), it has been proposed that simultaneous expression of two *VSG* genes is unstable and the induction of high levels of expression of a second *VSG* from a ribosomal locus results in transcriptional attenuation of the BES *VSG* (48). Upon selection for activation of two BESs, both *VSGs* colocalize to a single transcription compartment, where they undergo dynamic transcriptional switches between the two BESs, with monoallelic expression rapidly restored upon the removal of drug selection (42). The occupation of two BESs within a single ESB is thought to be energetically unfavorable (42), and attempts to select for the simultaneous activation of three BESs were unsuccessful (49).

The *VSG* transcript is essential for cell survival, and knockdown triggers precytokinesis cell cycle arrest (50) and global translation arrest (45). This lethal phenotype is rescued by VSG switching (50) or the expression of a second *VSG* (45). Consistent with this, a DSB introduced in the actively transcribed BES is a highly toxic lesion that results in over 95% cell mortality and all of the DSB survivors having switched the active VSG (26, 34). Here, we asked whether the toxicity of a BES DSB could be rescued by the expression of a second *VSG* and if this modified the *VSG* switching pathway used. We established a cell line in which monoallelic *VSG* expression is disrupted by the stable expression of a second telomeric *VSG* from a ribosomal DNA (rRNA gene) locus, resulting in the presence of both the ectopic and native VSGs at the cell surface. We found that using a targeted DSB in the active BES to trigger a VSG switch, the DNA sequence of the ectopic *VSG* is lost, despite it being located at a distinct genomic locus. Deletion of the ectopic *VSG* is transcription dependent and does not affect the number or the variety of genomic templates used for BES repair. We propose that monoallelic expression is strictly reinforced during antigenic variation, preventing multiple VSG switching events occurring at once, which would compromise parasite survival.

## RESULTS

### Expression of a second *VSG* does not rescue the toxicity of a DSB at the active BES.

**FIG 1:**
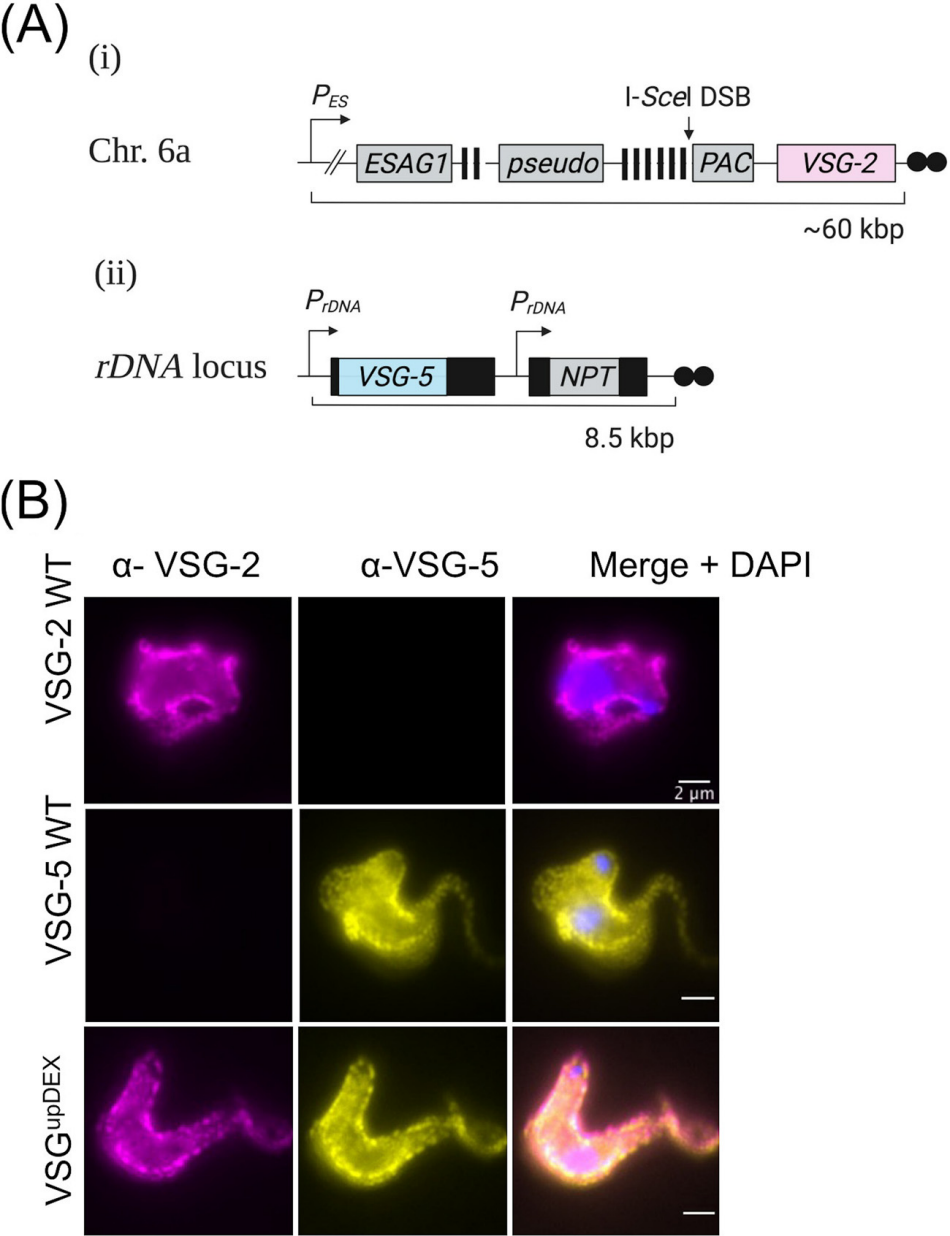
Two *VSG*s are stably expressed at the parasite cell surface. (A, i) Schematic of the VSG^up^ cell line, showing BES1 on chromosome 6a. An I-*Sce*I meganuclease recognition site (I-*Sce*I DSB) is inserted upstream of the actively expressed *VSG-2*. (A, ii) Schematic of the p^5^NTMF reporter construct that is integrated in a single-crossover event at an rDNA promoter to generate the VSG^upDEX^ cell line. mRNA-processing sequences are shown as black boxes. *VSG-5* is flanked by the procyclin 5′ UTR and 896 bp of the *VSG-2* downstream processing sequence. *NPT* is flanked by 5′ and 3′ aldolase-processing sequences. *P_ES_*, native BES1 promoter, striped boxes, 70-bp repetitive sequence; *PAC*, puromycin *N*-acetyltransferase resistance gene; *P_rDNA_*, ribosomal DNA promoter, *NPT*, neomycin phosphotransferase resistance gene; black circles, telomeric sequence. Figure created with BioRender.com. (B) Immunofluorescence analysis of surface VSGs using antibodies specific to VSG-2 and VSG-5, VSG-2 and VSG-5 wild-type (WT) single expressing cell lines are controls. Scale bars, 2 μm.

**FIG 2:**
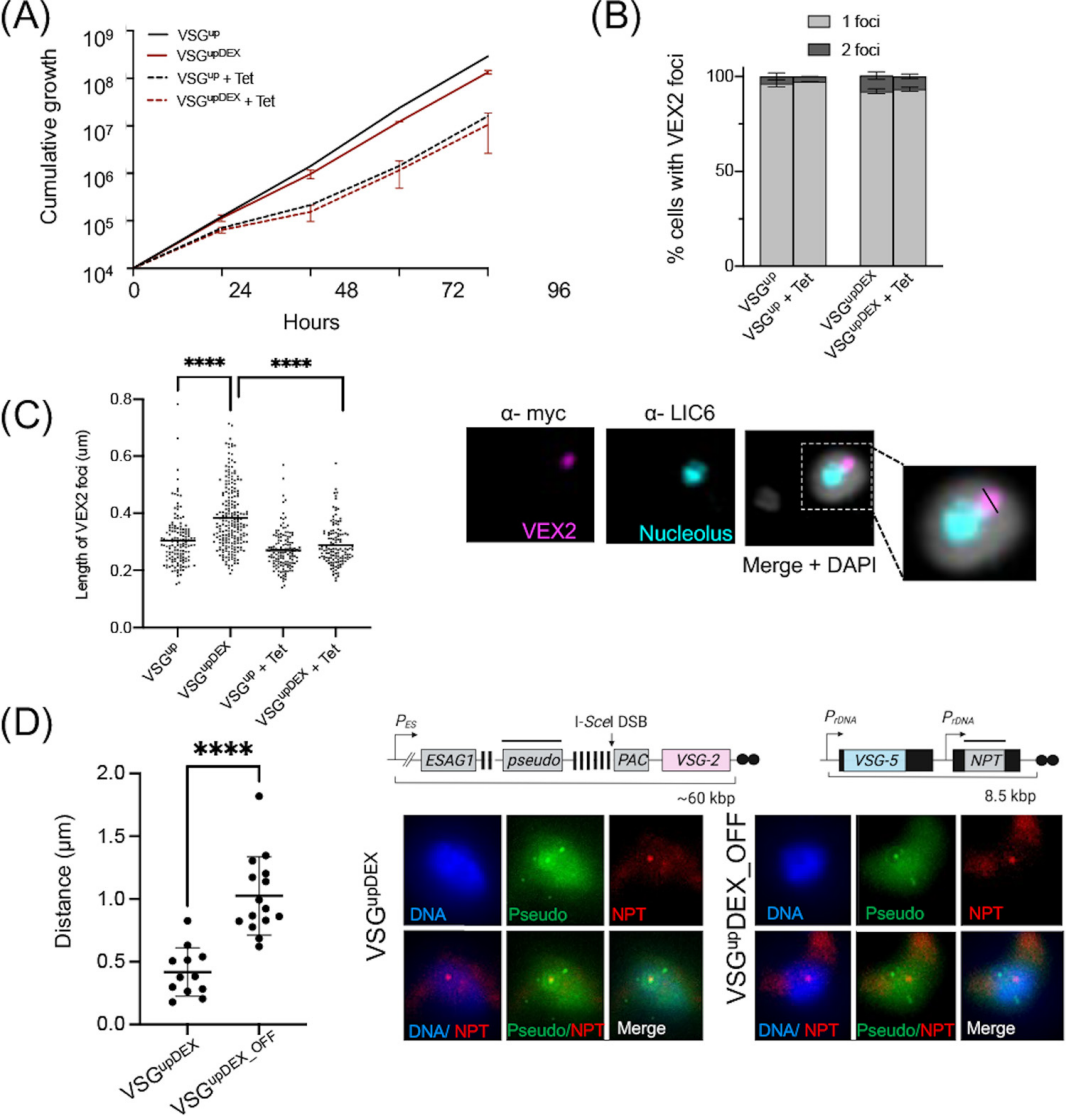
Expression of ectopic *VSG-5* results in an increase in the size of VEX2 foci. (A) Cumulative growth of VSG^up^ and VSG^upDEX^ cell lines. Error bars represent standard deviation between a single induction in a pair of biological clones. Dashed lines indicate growth under DSB-inducing conditions (+Tet). (B) Percentage of cells with one or two VEX2 foci in the VSG^up^ and VSG^upDEX^ cell lines. Cells gown under either uninduced conditions or DSB-inducing conditions for 7 days (+Tet) were scored for 1 or 2 VEX2 foci. Only cells in the G_1_ or S phase were counted. For the VSG^up^ cell line, error bars represent standard deviation between a pair of technical replicates. For the VSG^upDEX^ cell line, error bars represent standard deviation between a pair of biological clones. *n *> 100 cells for each condition. (C) VEX2 foci length (μm) in VSG^up^ and VSG^upDEX^ cell lines. Individual black circles represent a single measurement in a G_1_- or S-phase cell. +Tet indicates growth under DSB-inducing conditions for 7 days. Horizontal bar represents the mean. Significance was calculated using an unpaired *t* test; ****, *P < *0.0001. Righthand panel shows representative IFA image of an uninduced VSG^upDEX^. Scale bar, 2 μm. Inset shows a ×2 to ×3 magnification of the nucleus with black line to represent an example of the measurements made. (D, Left) Quantification of the distance between Pseudo and NPT foci in the VSG^upDEX^ and VSG^upDEX_OFF^ cell lines. *n* = 12 and *n* = 15 cells counted for the VSG^upDEX^ and VSG^upDEX_OFF^ cell lines, respectively; the average value is shown with a horizontal bar ± SD, and the significance of colocalization as ****, *P < *0.0001. Images were randomized to allow for blind counting. (D, Right) Schematics of BES1 and p^5^NTMF reporter with a black bar representing the DNA FISH probe. Representative images of the DNA FISH from the VSG^upDEX^ and VSG^upDEX_OFF^ cell lines.

The established VSG^up^ cell line facilitates the induction of a specific DSB adjacent to the 70-bp repeats in BES1 (25, 26), from which *VSG-2* (also known as *VSG221* [5]) is actively expressed (Fig. 1Ai). Tetracycline (Tet)-inducible expression of the I-*Sce*I meganuclease cleaves an 18-bp recognition site adjacent to the 70-bp repeats upstream of the *VSG* in BES1 and is highly deleterious to the cell population, with only 5% of the population able to survive. Among the population of DSB survivors, there is a 5,000-fold increase in the rate of VSG switching (26). Previous reports have shown that the simultaneous expression of two *VSGs* does not disrupt T. brucei parasite growth (42, 43, 46, 51) and, therefore, to determine whether the expression of a second copy of a *VSG* gene could rescue the toxic effect of a DSB at the active BES, we introduced an ectopic copy of *VSG-5* into the VSG^up^ cell line at a ribosomal promoter using the p^5^NTMF construct (19). The *VSG-5* expression construct contains a 200-bp telomere seed, and the single crossover event at the ribosomal promoter therefore generates a *de novo* telomere (19, 52) (Fig. 1Aii). Approximately 20% of the transformed clones show high-level *VSG-5* expression (Fig. S1A in the supplemental material), and the presence of both VSG-2 and VSG-5 at the cell surface was confirmed by immunofluorescence assay (IFA) (Fig. 1B). Clones expressing high levels of *VSG-5* are Double EXpressing (DEX) and are referred to as VSG^upDEX^. Clones where VSG-5 expression was not detected are referred to as VSG^upDEX_OFF^. The p^5^NTMF construct contains an NPT gene for selection and, therefore, to determine whether *VSG-5* expression is stable, the VSG^upDEX^ clones were grown in the absence of *NPT* selection for 7 days by removal of the G418 drug from the growth medium. Protein analysis revealed that both *VSG-5* and *NPT* are expressed in the absence of selection pressure, indicating that the expression of ectopic *VSG-5* is stable (Fig. S1B). We then monitored the rate of parasite proliferation in the VSG^up^ and VSG^upDEX^ cell lines under both DSB-inducing and -noninducing conditions. We did not observe growth differences (Fig. 2A, solid lines), nor an increase in survival of VSG^upDEX^ cells (Fig. 2A, dashed lines), indicating that the expression of a second *VSG* alone does not ameliorate the toxicity of a BES DSB.

10.1128/mbio.03847-21.1 FIG S1 *VSG-5* is stably expressed from a ribosomal locus. (A) Coomassie gel showing 10 VSG^upDEX^ clones. Clones 1 and 7 show a band at the expected size for VSG-5, highlighted with a white asterisk. (B) VSG-5 protein expression is stable in the absence of drug selection pressure. Western blot analysis showing proteins harvested from a pair of VSG^upDEX^ clones grown in the presence (C1 and C2) and absence of G418 for 7 days (C1^−G418^ and C2^−G418^). Staining for H3 and EIF1α serves as loading controls. Download FIG S1, TIF file, 2.1 MB. Copyright © 2022 McLaughlin et al. 2022 McLaughlin et al. https://creativecommons.org/licenses/by/4.0/ This content is distributed under the terms of the Creative Commons Attribution 4.0 International license.

The site of *VSG* transcription is marked by an extranucleolar pool of RNA Pol I called the ESB (9, 12) and the VEX complex (19, 20). VEX2 forms a single focus in the nucleus that is coincident with the actively expressed BES (20). We therefore asked whether the ectopically expressed *VSG-5* was able to recruit VEX2 and whether the number of foci was consistent between the VSG^up^ and VSG^upDEX^. One allele of VEX2 was natively tagged (20) and the nucleolus marked using the L1C6 antibody (53). VSG^up^ and VSG^upDEX^ cells were scored for one or two VEX2 foci in G_1_- and S-phase cells (Fig. 2B). For VSG^up^ and VSG^upDEX^, 96% and 92% of cells, respectively, had one VEX2 focus prior to DSB induction, and this did not change following a DSB (Fig. 2B). Two active BESs have been shown to colocalize to a single ESB (42), and the identification of a single VEX2 focus in the large majority of VSG^upDEX^ cells suggests that ectopic *VSG-5* expressed from an rDNA locus occupies the same transcription compartment as BES1. During our experiments, we observed that the VEX2 foci appeared larger in the VSG^upDEX^ cells, which led us to carry out measurements of the length of the VEX2 focus in both cell lines. In the VSG^up^ cell line, the average VEX2 focus length was 0.30 μm, while in the VSG^upDEX^ cells, it was significantly larger, with an average length of 0.38 μm (*P < *0.0001) (Fig. 2C). We then assessed the size of the VEX2 focus following a DSB. We found that the length of the VEX2 focus in the VSG^upDEX^ cell line was reduced to an average length of 0.29 μm, which is not significantly different from the uninduced VSG^upDEX^ and is similar to the VSG^up^ cell line. This indicates that in the VSG^upDEX^ cell line, the VEX2 *VSG* transcription compartment is larger, possibly as a result of accommodating the transcription of two *VSGs*, but this is not maintained following a DSB and subsequent repair. To directly test this, we performed DNA fluorescent *in situ* hybridization (FISH) using probes to the *Pseudo* gene, found in the active BES, and the *NPT* gene in the p^5^NTMF construct. In order to compare the position of the *NPT* gene, and thus the ectopic *VSG*, in the nucleus when actively expressed and silenced, we examined both the VSG^upDEX^ and VSG^upDEX_OFF^ cell lines. We found that in the VSG^upDEX^ cells, the *Pseudo* and *NPT* foci were an average distance of 0.4 ± 0.19 μm apart, which was significantly closer in proximity than the VSG^upDEX_OFF^ cells, where the foci were 1.02 ± 0.312 μm apart (Fig. 2D). We also noted that in the VSG^upDEX_OFF^ cells, the NPT focus was seen within the nucleolus, as expected given that integration of this construct is at a ribosomal promoter (Fig. S2). This, along with the increase in the size of the VEX2 foci, indicated that the ectopic *VSG-5* copy occupies the same transcription compartment as BES1 (Fig. 2D).

10.1128/mbio.03847-21.2 FIG S2 Ectopic *VSG-5* is localized to the nucleolus when silent. Representative images of the NPT DNA FISH in the VSG^upDEX_OFF^ cell line. Nucleolus is the region with the less intense DAPI staining. N, Nucleus; K, kinetoplast; No, nucleolus. Download FIG S2, TIF file, 2.1 MB. Copyright © 2022 McLaughlin et al. 2022 McLaughlin et al. https://creativecommons.org/licenses/by/4.0/ This content is distributed under the terms of the Creative Commons Attribution 4.0 International license.

### VSG-2 and VSG-5 are lost from the cell surface following a DSB.

**FIG 3:**
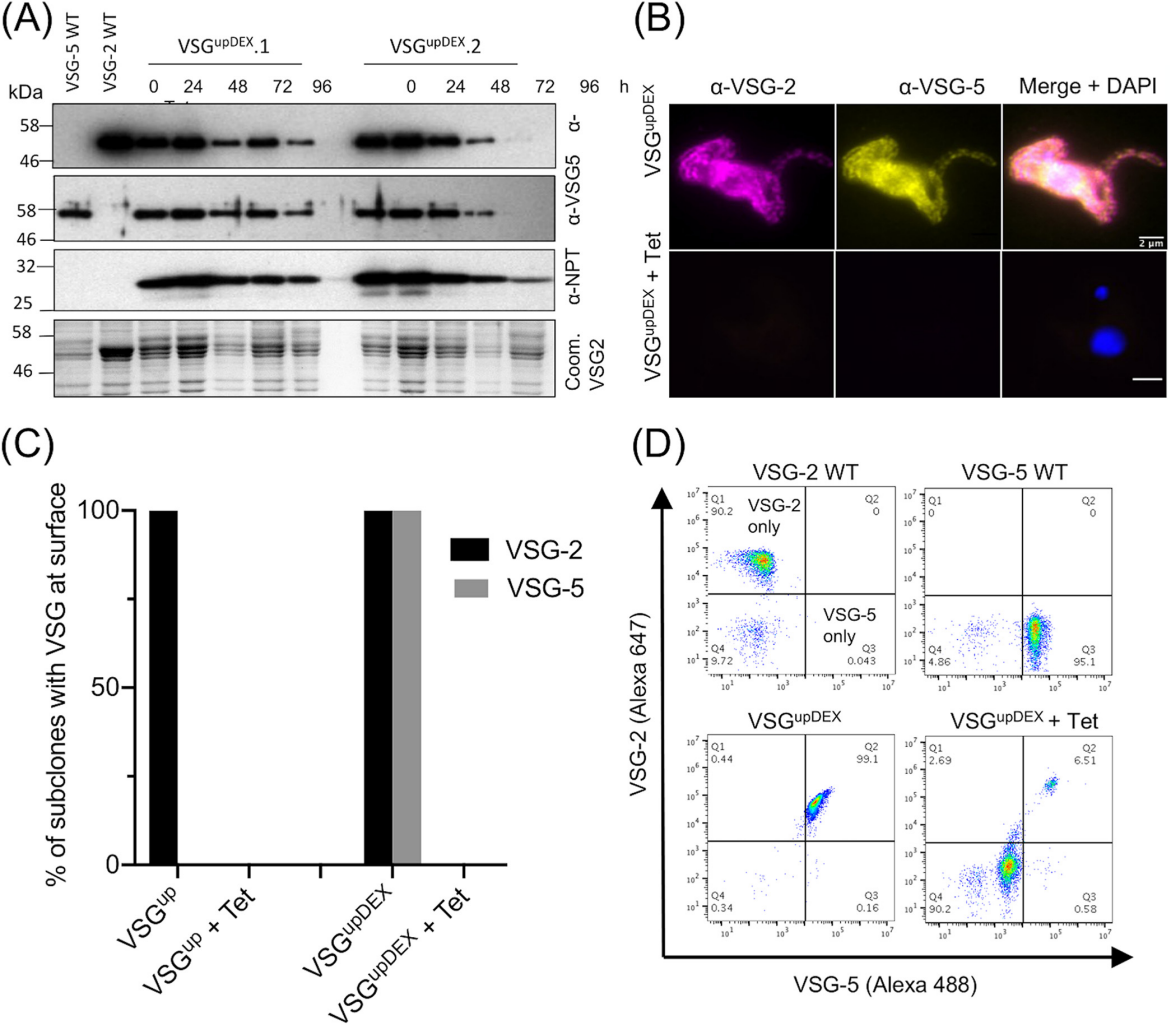
VSG-2 and VSG-5 are lost from the cell surface following a DSB. (A) Western blot analysis of protein extracted from VSG^upDEX^ clones over 96 h following induction of an I-*Sce*I DSB. VSG-2 and VSG-5 WT single expressing cell lines are controls. Protein extracts were probed using antibodies specific to VSG-2, VSG-5. and NPT. The bottom panel is Coomassie stained and serves as a loading control and also reveals the major VSGs. (B) IFA of VSG^upDEX^ using antibodies specific for VSG-2 and VSG-5. +Tet indicates 7 days DSB induction. (C) Immunofluorescence assay showing the presence of VSG-2 and VSG-5 at the cell surface of VSG^upDEX^ subclones. Uninduced subclones, *n* = 5; induced subclones (+Tet), *n* = 20. (D) Analysis of VSG^upDEX^ surface VSGs 0 and 7 days post-DSB induction by flow cytometry. Quantitation of the abundance of VSG-5 stained with Alexa Fluor 488 secondary antibody is shown on the *x* axis, while VSG-2 stained with Alexa Fluor 647 secondary antibody is shown on the y- axis. (D, Top) Single expressing WT cell lines. (D, Bottom) VSG^upDEX^ uninduced is shown on the left-hand side, and induced with tetracycline for 7 days (+Tet) is shown on the right-hand side.

Given the increase and subsequent decrease in the size of the VEX2 focus in the VSG^upDEX^, we assessed the expression of VSG-2 and VSG-5 protein following a DSB. An I-*Sce*I DSB was induced in the VSG^upDEX^ cell lines in the absence of any drug selection. Protein analysis revealed that VSG-2 decreased over 96 h following a DSB (Fig. 3A), which is expected, as the *VSG-2* gene is located downstream of the DSB site (Fig. 1Ai) and lost following a break (26). Protein analysis of both VSG-5 and NPT also showed a decrease over the course of the induction (Fig. 3A), suggesting that VSG-5 is not maintained at the cell surface following a DSB at the active BES. We next examined parasite populations for surface VSGs by immunofluorescence assay. As it takes approximately 4.5 days to completely replace the VSG coat (46), we analyzed cells at 7 days post-DSB induction. Neither VSG-2 nor VSG-5 were detected in the population (Fig. 3B). We then wanted to assess this finding in individual subclones. For this, we generated a panel of five uninduced subclones and 20 DSB repaired subclones for each of the VSG^up^ and VSG^upDEX^ cell lines. DSB induction was confirmed in all induced subclones by sensitivity to puromycin. The puromycin resistance gene is located downstream of the DSB site in the VSG^up^ cell line and is either lost or disrupted following a DSB. All induced subclones were puromycin sensitive, while all uninduced subclones were puromycin resistant, confirming I-*Sce*I cleavage. While both VSG-2 and VSG-5 were detected at the surface of all uninduced subclones, both were lost from the surface of all 20 DSB-induced subclones (Fig. 3C). This was confirmed by fluorescence-activated cell sorting (FACS) of a population at 0 and 7 days post-DSB. In the uninduced cells, both VSG-2 and VSG-5 are detected at the surface of 99.1% of cells, and this was reduced to 6.5% of cells 7 days post-DSB induction (Fig. 3D), with a second clone behaving in a similar manner (Fig. S3). The remaining 6.5% VSG-2/VSG-5-positive cells most likely represent those not cleaved by I-*Sce*I. This suggests that a DSB in BES1 results in the loss of VSG-5 from the cell surface, despite the *VSG-5* gene being located at a distinct locus to BES1.

10.1128/mbio.03847-21.3 FIG S3 Flow cytometry analysis of a second VSG^upDEX^ clone. Quantitation of the abundance of VSG-5 stained with Alexa Fluor 488 secondary antibody is shown on the *x* axis, while VSG-2 stained with Alexa Fluor 647 secondary antibody are shown on the *y* axis. The left-hand panel shows analysis of the uninduced clone, and the right-hand panel shows analysis 7 days post-DSB induction (+Tet). Download FIG S3, TIF file, 2.1 MB. Copyright © 2022 McLaughlin et al. 2022 McLaughlin et al. https://creativecommons.org/licenses/by/4.0/ This content is distributed under the terms of the Creative Commons Attribution 4.0 International license.

### Ectopic *VSG-5* is lost from the genome following a DSB.

**FIG 4:**
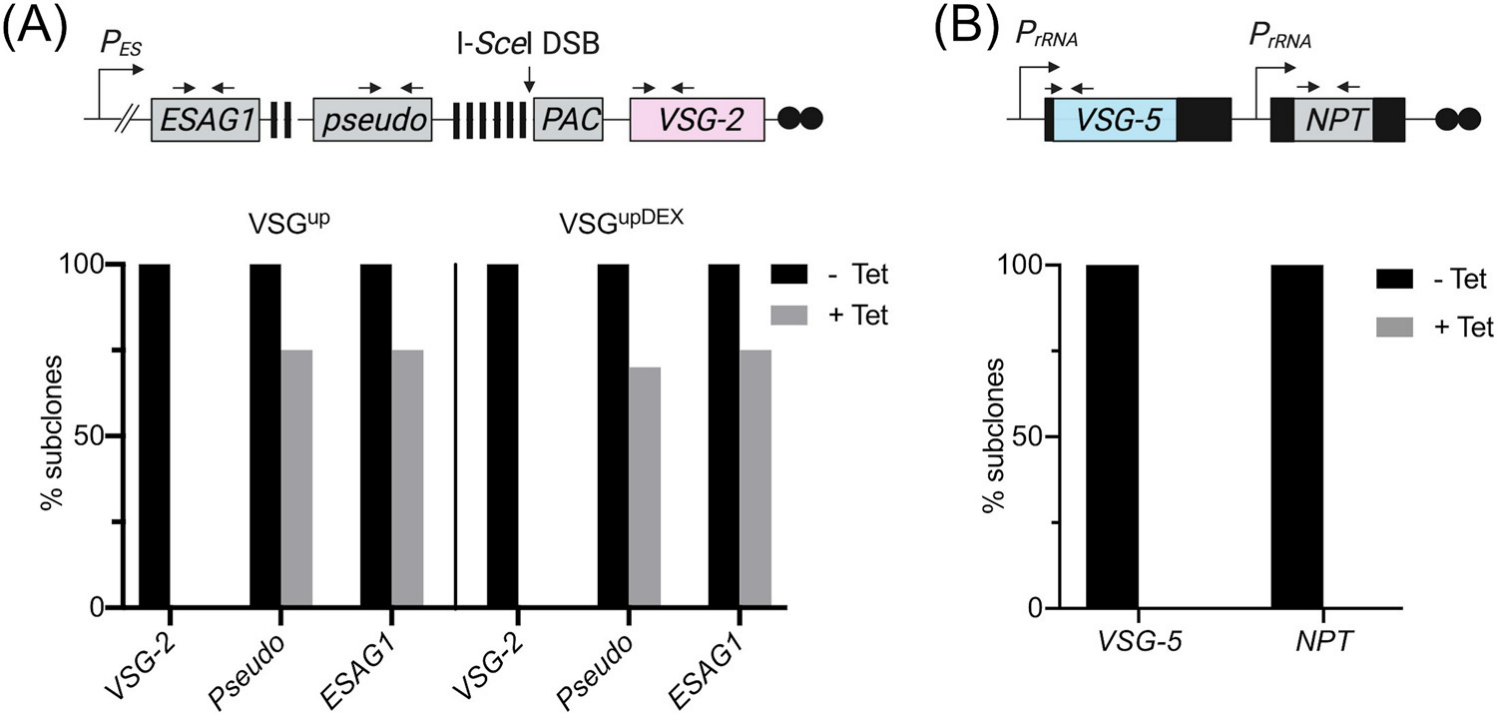
The ectopic copy of *VSG-5* is lost following a DSB. (A) PCR analysis of repair at the active BES of VSG^up^ and VSG^upDEX^ subclones. (A, Top) Schematic of the modified BES1 with black arrows to show the position of primer binding sites used for subclone analysis; all other details as in Fig. 1Ai. (A, Bottom) PCR analysis of VSG^up^ and VSG^upDEX^ subclones to detect the presence or absence of *VSG-2*, *pseudo*, and *ESAG1.* Uninduced subclones (−Tet), *n* = 5; induced subclones (+Tet) *n* = 20. (B, Top) Schematic with primers used to specifically amplify *VSG-5* and *NPT* shown in black arrows; all other details as in Fig. 1Aii. (B, Bottom) PCR analysis of *NPT* and *VSG-5* in VSG^upDEX^ subclones. Uninduced subclones (−Tet), *n* = 5; induced subclones (+Tet), *n* = 20.

The concurrent reduction in VSG-2 and VSG-5 at the trypanosome surface following a BES DSB led us to ask how the VSG^upDEX^ cells repaired the I-*Sce*I DSB. It has been shown that a DSB in the active BES is repaired predominantly via gene conversion using the 70-bp repetitive sequences for homology (25, 26, 54). To map individual repair events in the VSG^up^ and VSG^upDEX^ cell lines, we used our cohort of subclones (5 uninduced and 20 induced survivor subclones for each cell line) and a series of established PCR assays to assess the presence or absence of several marker genes in BES1 (26, 55). Using primers specific to *VSG-2*, we found that for both VSG^up^ and VSG^upDEX^, all uninduced subclones (*n* = 5) were *VSG-2* positive by PCR, and all induced subclones (*n* = 20) were *VSG-2* negative (Fig. 4A; Fig. S4A and B), supporting both the IFA and FACS data showing loss of the protein in VSG^upDEX^ (Fig. 3B and C). BES1 contains two blocks of 70-bp repeats; the first block is located immediately upstream of the *VSG*, and a second, smaller, 70-bp repeat region is found further upstream, flanked by the *pseudo* gene and *ESAG1* (Fig. 4A) (7). Cells that are PCR positive for *ESAG1* and *pseudo* are expected to have undergone GC using the 70-bp repeats directly upstream of *VSG-2* as a homologous template. If *pseudo* is lost but *ESAG1* preserved, repair has likely occurred by GC using the smaller block of 70-bp repeats. Subclones that have lost *ESAG1*, *pseudo*, and *VSG-2* have likely undergone BES loss. Following a VSG^up^ DSB, 75% of subclones (*n* = 15) retained both *ESAG1* and *pseudo* (Fig. 4A and Fig. S4A), in agreement with previous findings that the large block of 70-bp repeats immediately upstream of the DSB is favored for repair (25, 26). For VSG^upDEX^, the findings were notably similar, with 70% (*n* = 14) and 75% (*n* = 15) retaining *pseudo* and *ESAG1*, respectively (Fig. 4A and Fig. S4B). Six repaired VSG^upDEX^ subclones had lost *pseudo*, and 5 lost *ESAG1*, suggesting repair by recombination upstream of these genes or loss of the entire BES. The one induced subclone that was *ESAG1* positive but *pseudo* negative likely repaired the DSB via gene conversion with the smaller block of 70-bp repeats upstream of *pseudo* gene. Importantly, these analyses demonstrate that the expression of a second *VSG* does not disrupt the regions of homology used within the BES to repair the DSB.

10.1128/mbio.03847-21.4 FIG S4 PCR analysis of VSG^up^ (A) and VSG^upDEX^ (B) subclones. Analysis was carried out in 5 uninduced subclones (black text, lanes 1 to 5) and 20 induced subclones (red text, lanes 6 to 25). Band sizes expected for *VSG-2, pseudo*, and *ESAG1* PCR reactions are 527 bp, 550 bp, and 380 bp, respectively. (C) PCR assays to amplify *VSG-5* and *NPT* in VSG^upDEX^ subclones. Five uninduced subclones (black text, lanes 1 to 5) and 20 induced subclones (red text, lanes 6 to 25). Band sizes expected for *VSG-5* and *NPT* PCR reactions are 225 bp and 515 bp, respectively. Download FIG S4, TIF file, 2.1 MB. Copyright © 2022 McLaughlin et al. 2022 McLaughlin et al. https://creativecommons.org/licenses/by/4.0/ This content is distributed under the terms of the Creative Commons Attribution 4.0 International license.

We next looked for the presence or absence of the ectopic expression construct genes *VSG-5* and *NPT* in the VSG^upDEX^ subclones. The T. brucei genome contains three copies of *VSG-5* (6), and amplification of the ectopic copy of *VSG-5* was achieved using primers specific to the procyclin 5′ untranslated region (UTR) and *VSG-5* (Fig. 4B). In this construct, the procyclin 5′ UTR is used for processing. Unexpectedly, all VSG^upDEX^-induced subclones (*n* = 20) were negative for both *VSG-5* and *NPT* (Fig. 4B and Fig. S4C). This indicates that the loss of *VSG-5* at the cell surface is accompanied by loss of the DNA sequence. To validate loss of these sequences, we carried out whole-genome sequencing on 2 uninduced and 4 induced VSG^upDEX^ subclones. Reads aligning uniquely to *NPT* were not observed in any of the four induced subclones (Fig. S5), consistent with the findings of the PCR assay. We were unable to identify reads specifically mapping to ectopic *VSG-5* using short paired-end reads. Together, these data demonstrate that the loss of VSG-5 and NPT protein is a result of loss of the DNA sequence.

10.1128/mbio.03847-21.5 FIG S5 Whole-genome sequencing of VSG^upDEX^ subclones. (A) Sequences of two uninduced VSG^upDEX^ subclones and four induced subclones are aligned to ectopic *VSG-5-NPT,* shown in the schematic at the bottom panel. In the schematic, black boxes represent the ribosomal promoter, and the start position is indicated with a black arrow. Gray boxes are UTR sequences, with *VSG-5* flanked by the 5′ UTR of EP procyclin and 3′ by 896 bp of *VSG-2* downstream processing sequence. The *NPT* is flanked by 5′ and 3′ aldolase processing sequences. Sequencing was carried out by paired-end 100-bp sequencing (BGI-seq). Reads were aligned with a mapping quality (MAPQ) of 1. Download FIG S5, TIF file, 2.1 MB. Copyright © 2022 McLaughlin et al. 2022 McLaughlin et al. https://creativecommons.org/licenses/by/4.0/ This content is distributed under the terms of the Creative Commons Attribution 4.0 International license.

The *NPT* resistance cassette used for integration of the *VSG-5* expression construct allowed us to test whether we could retain the ectopic *VSG-5* copy using G418 selection. We saw no difference between the VSG^up^, VSG^upDEX^, or VSG^upDEX_OFF^ cells grown under DSB-inducing conditions and selection using 2 μg/mL of G418 for 7 days (Fig. S6A). We expect the VSG^upDEX_OFF^ cells to behave similarly to the VSG^up^ cells, as they contain the *VSG-5* ectopic construct, but it is not transcribed. The presence of VSG-2 and VSG-5 at the cell surface was then examined by IFA. In the uninduced cells, 100% of the cell population expressed only VSG-2 in VSG^up^ and VSG^upDEX_OFF^, and 100% of the VSG^upDEX^ population expressed both VSG-2 and VSG-5 on their surface (Fig. S6B). Following DSB induction in VSG^up^, 95% of cells switched. In the VSG^upDEX^ and VSG^upDEX_OFF^ cells, induction and selection with G418 resulted in 96% and 3% of cell switching, respectively (Fig. S6B). We then assessed a panel of subclones to determine the efficiency of I-*Sce*I cutting and DSB repair in the cells. Out of 30 induced subclones tested, 29 were puromycin resistant (Fig. S6C), indicating that the majority of the subclones were not subject to an I-*Sce*I DSB. Given that the I-*Sce*I meganuclease has over 95% efficiency of cutting (56), this suggests that by using G418 selection, we have applied a strong selective pressure for uncut cells. The single puromycin-sensitive subclone was VSG-2 and VSG-5 positive by IFA (data not shown) and has likely repaired the DSB using short regions of homology for imperfect repair, resulting in disruption of the puromycin open reading frame but preserving *VSG-2* in BES1. We were unable to identify any cells that had undergone GC to repair the DSB while maintaining VSG-5 at the cell surface. This indicates that it is highly unfavorable to undergo *VSG* switching while a second VSG is present at the cell surface.

10.1128/mbio.03847-21.6 FIG S6 Retention of ectopic *VSG-5* cannot be selected for using drug selection pressure. (A) Cumulative growth of VSG^up^, VSG^upDEX^, and VSG^upDEX_OFF^ cell lines. Error bars represent standard deviation between a replicate induction. A pair of biological clones was used for the VSG^upDEX^ and VSG^upDEX_OFF^ cell lines. Dashed lines indicate growth under DSB-inducing conditions (+Tet) with G418 selection in the VSG^upDEX^ and VSG^upDEX_OFF^ cell lines. (B) Percentage of cells in VSG^up^, VSG^upDEX^, and VSG^upDEX_OFF^ positive for VSG-2 and VSG-5 7 days following DSB induction and selection with G418 as measured by IFA; *n* > 100 cells. (C) Puromycin resistance among 30 VSG^upDEX^ subclones that have been DSB induced and selected using G418. Download FIG S6, TIF file, 2.1 MB. Copyright © 2022 McLaughlin et al. 2022 McLaughlin et al. https://creativecommons.org/licenses/by/4.0/ This content is distributed under the terms of the Creative Commons Attribution 4.0 International license.

### Loss of *VSG-5* is dependent on transcription.

**FIG 5:**
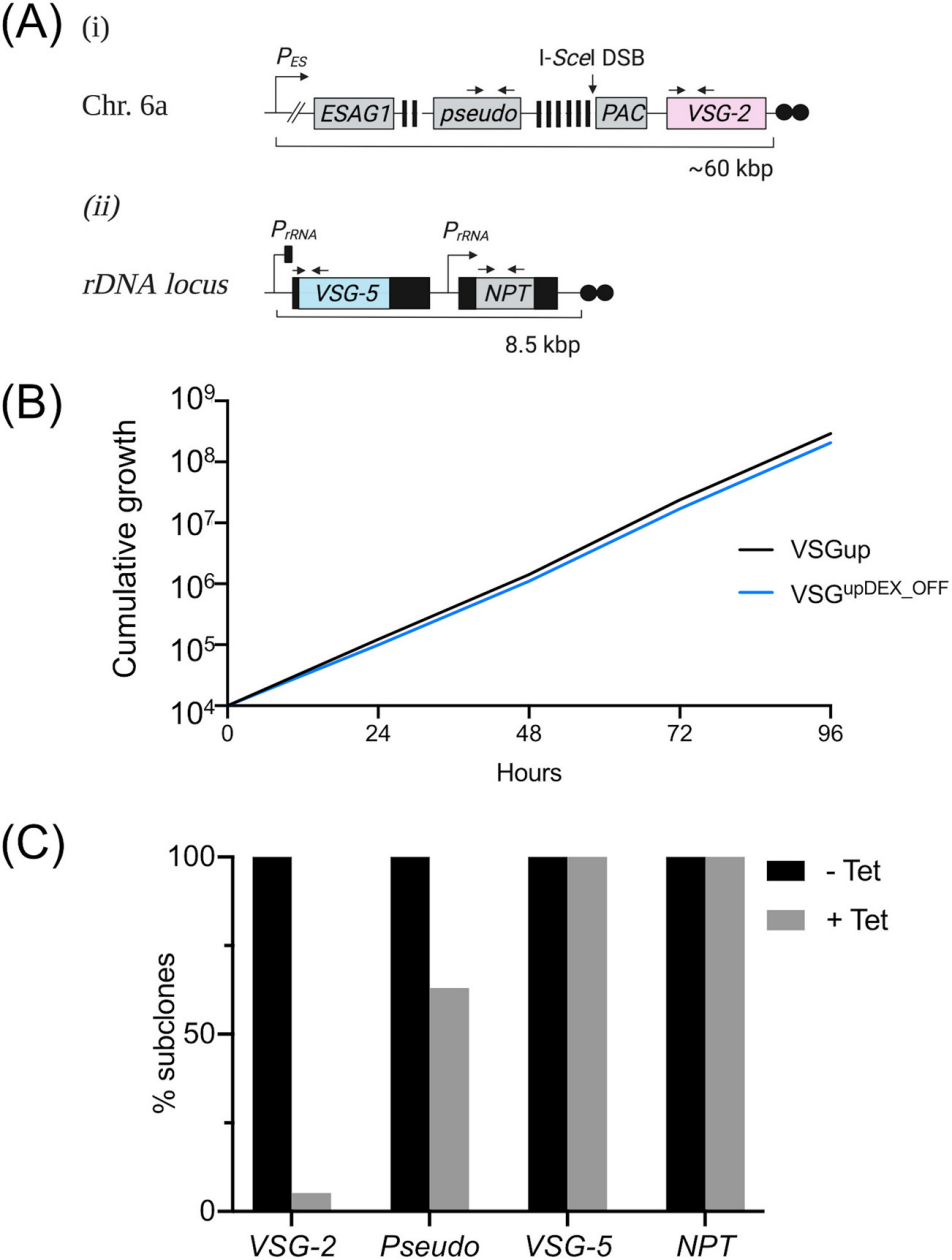
Loss of ectopic *VSG-5* is dependent on transcription. (A) Schematic of the VSG^upDEX_OFF^ cell line setup. (i) Modified BES1; details as in Fig. 1Ai; (ii) the transcriptionally silenced p^5^NTMF construct. The ribosomal promoter upstream of *VSG-5* is silenced, as indicated by a black rectangular bar. All other details as in Fig. 1Aii. (B) Cumulative growth of the VSG^upDEX_OFF^ cell line over 96 h. (C) PCR analysis of VSG^upDEX_OFF^ subclones. Primer binding sites for PCRs are shown as black arrows in Fig. 6A. −Tet, *n* = 5; +Tet, *n* = 20.

Loss of the ectopic VSG*-5* expression construct following a DSB was unexpected, and we hypothesized that its transcriptional state could be an influence. Of the 10 VSG^upDEX^ cell lines we originally generated, only 20% expressed *VSG-5* (Fig. S1); we therefore selected 2 clones where *VSG-5* is silenced and *VSG-2* is the singly dominant *VSG* expressed. We refer to this cell line as VSG^upDEX_OFF^ (Fig. 5A and Fig. S7A). As in the VSG^upDEX^ cell line, cell growth was not affected in VSG^upDEX_OFF^, demonstrating that growth is not affected by the integration of the p^5^NTMF construct (Fig. 5B). We then generated a cohort of 5 uninduced and 20 induced VSG^upDEX_OFF^ subclones for further analysis. All induced subclones were puromycin sensitive, indicating efficient cutting by I-*Sce*I. Using our PCR assays, we found that 95% of the induced VSG^upDEX_OFF^ subclones had lost *VSG-2*, and 65% had retained the *pseudo* gene (Fig. 5C and Fig. S7B), indicating that repair using the 70-bp repeats for recombination dominated. In striking contrast to the VSG^upDEX^ cell line, all 20 VSG^upDEX_OFF^ subclones retained both *VSG-5* and *NPT* following DSB induction (Fig. 5C and Fig. S7B). This demonstrates that the loss of *VSG-5* and *NPT* in the VSG^upDEX^ cell line is transcription dependent.

10.1128/mbio.03847-21.7 FIG S7 *VSG-5* is silenced in the VSG^upDEX_OFF^ cell line. (A) Western blot analysis of *VSG-2* and *VSG-5* expression in the VSG^upDEX^ and VSG^upDEX_OFF^ cell lines. DEX.1 and DEX.2 represent a pair of VSG^upDEX^ biological clones; DEX^OFF^ is a VSG^upDEX_OFF^ clone. Protein extracts were probed with antibodies specific to VSG-2 (above) or VSG-5 (below). H3 serves as a loading control. (B) PCR reactions for repair at the active BES in VSG^upDEX_OFF^ clones. Analysis was carried out in 5 uninduced subclones (black text, lanes 1 to 5) and 20 induced subclones (red text, lanes 6 to 24). Data for one induced subclone not shown. Expected band sizes for *VSG-2, pseudo*, *VSG-5*, and *NPT* PCR reactions are 527 bp, 550 bp, 225 bp, and 515 bp, respectively. Download FIG S7, TIF file, 2.1 MB. Copyright © 2022 McLaughlin et al. 2022 McLaughlin et al. https://creativecommons.org/licenses/by/4.0/ This content is distributed under the terms of the Creative Commons Attribution 4.0 International license.

### The accumulation of single-stranded DNA is delayed in VSG^upDEX^ cells.

**FIG 6:**
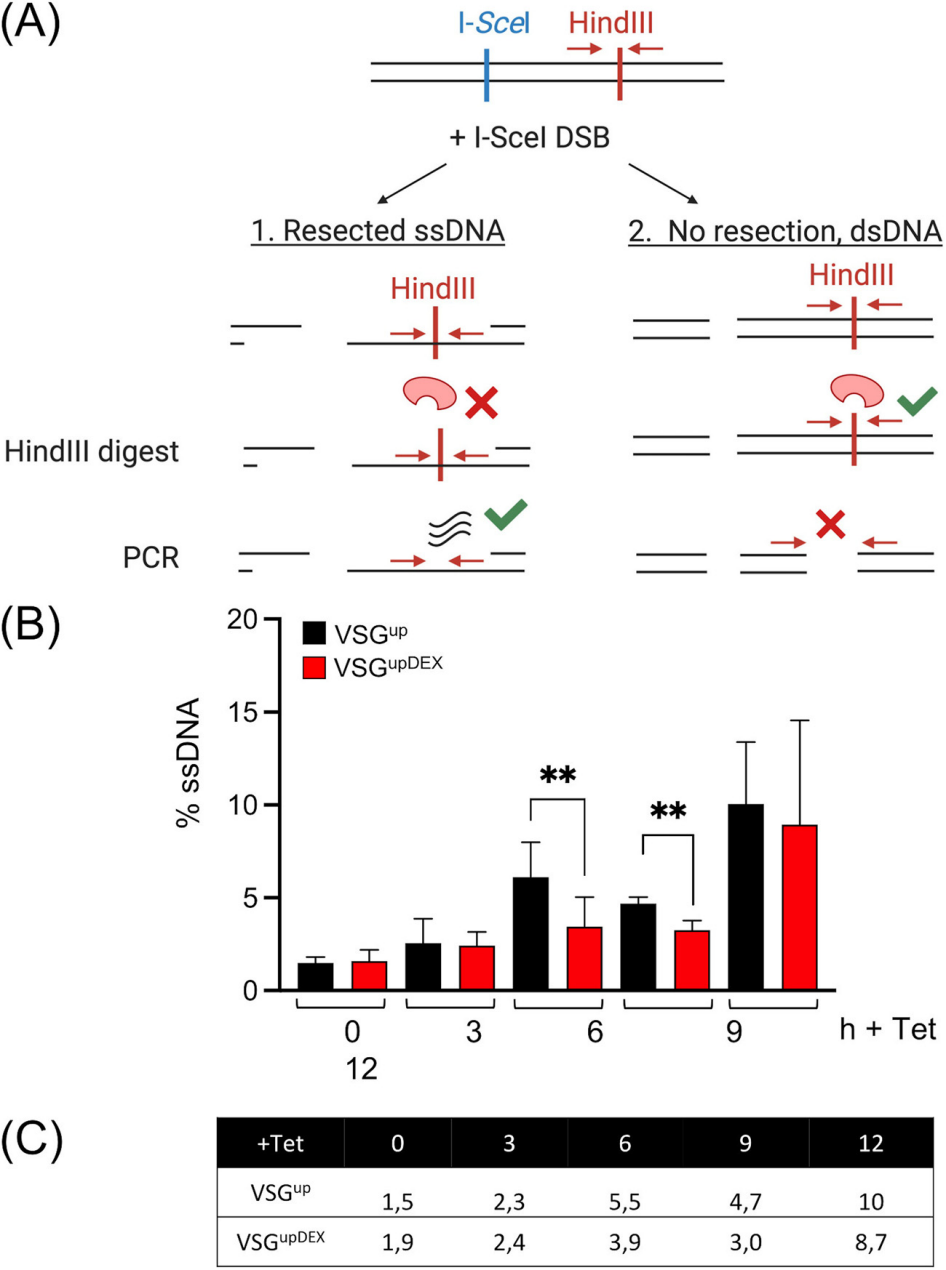
The expression of a second *VSG* disrupts the timing of repair at the active BES. (A) Schematic of the quantitative assay used to measure ssDNA resection. Black lines represent genomic DNA harvested from parasites at different time points following I-*Sce*I induction. The I-*Sce*I recognition site in BES1 is shown as a blue bar and the HindIII digestion site with a red bar. Pathway 1 shows the outcome if the DNA adjacent to the I-*Sce*I restriction site is single stranded. HindIII digestion will not proceed, as shown with a red cross. PCR amplification of either side of the HindIII site, demonstrated with red arrows, will produce a product as the template is intact (green tick). Pathway 2 shows the outcome if the DNA adjacent to the I-*Sce*I restriction site is double stranded. HindIII will digest (green tick), and the PCR will not yield a product, as the template has been destroyed (red cross). Mock digestion with HindIII is used as a control. Figure created with BioRender.com (B) Quantification of DNA end resection, measured in VSG^up^ and VSG^upDEX^ cell lines 0, 3, 6, 9, and 12 h following induction of a DSB. The VSG^up^ error bars represent standard deviation between a pair of technical replicates, while the VSG^upDEX^ error bars represent standard deviation between a pair of biological clones. The qPCR assay was performed in triplicate. Significance was calculated using an unpaired *t* test; **, *P < *0.001. (C) Table summarizing the percentage of ssDNA in each cell line following induction of a DSB.

Following a DSB, resection proceeds rapidly to facilitate repair. Given the loss of both the native *VSG-2* sequence and the ectopic *VSG-5* sequence, we next investigated whether the timing of the DNA damage response at the active BES was disrupted. A quantitative resection assay was employed (55, 57, 58) that makes use of a HindIII site within *VSG-2* to assess the timing of single-stranded DNA (ssDNA) accumulation at the active BES (Fig. 6A). In brief, genomic DNA was harvested at 0, 6, and 12 h post-DSB induction. The genomic DNA (gDNA) was then either digested with HindIII or mock digested. If the gDNA is single stranded (ssDNA) at the HindIII site, the restriction digest will be inhibited, and a PCR product will be amplified (Fig. 6A). If the DNA is double stranded, HindIII digestion will proceed, and the template for the PCR amplification is destroyed. Therefore, a PCR product will only be amplified if the originating gDNA is single stranded at the HindIII site. For each sample, the amount of PCR product is compared to that of an undigested “mock”-digested sample in which the template remains intact and a PCR product is amplified. For the VSG^up^ cell lines, we found that ssDNA increased over time following a DSB at 0, 3, 6, 9, and 12 h, respectively (Fig. 6B and C), in agreement with previously published data (26, 55). For VSG^upDEX^, the relative amount of ssDNA was significantly lower at 6 and 9 h post-DSB compared to VSG^up^ (*P < *0.0001) (Fig. 6B). By 12 h, the VSG^upDEX^ cell lines’ ssDNA was similar to that of VSG^up^ at the same time point. Therefore, the initial stages of DNA resection are delayed in VSG^upDEX^ cell line.

### Access to the *VSG* archive is not affected in the VSG^upDEX^ cells.

**FIG 7:**
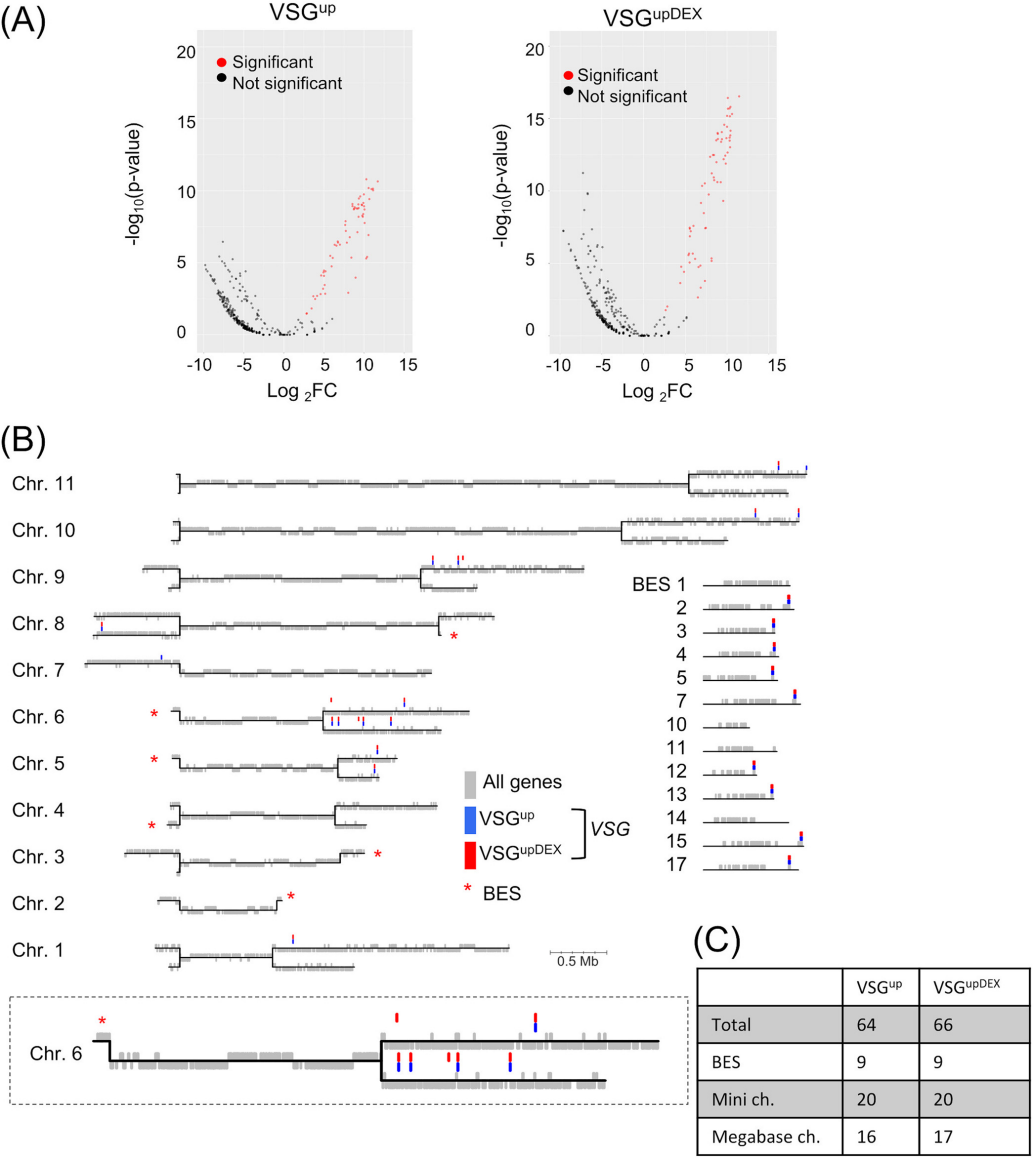
The expression of a second *VSG* does not disrupt VSG switching pathway. (A) Volcano plots showing the significantly enriched *VSG* genes identified by VSG-seq following a BES DSB in the VSG^up^ and VSG^upDEX^ cell lines. RNA was extracted 0 and 7 days following I-*Sce*I induction. Red circles represent genes that are significantly upregulated after 7 days DSB induction (log_2_FC > 2 and *P < *0.05), and black circles represent genes that are not significantly upregulated; *n* = 3. (B) Map of the T. brucei 427 genome showing the position of significantly upregulated *VSG* genes following a DSB. Megabase chromosomes are shown as black horizontal lines and genes as gray vertical lines. *VSG* genes that are significantly upregulated following a DSB are highlighted with blue bars for the VSG^up^ and red bars for VSG^upDEX^. BESs are highlighted with a red star on the genome map, and individual BESs are shown separately on the right-hand side. An enlarged version of chromosome 6 is shown below. (C) Table summarizing the position of enriched *VSG* genes identified by VSG-seq.

The transcription-dependent loss of *VSG-5* following a VSG switch indicates that there are stringent mechanisms at play to ensure that more than one VSG is not activated during VSG switching, preventing multiple switching events from occurring at once. Taken together with the initial delay in resection, which is critical to repair pathway choice, we wanted to determine whether the cohort *VSG* genes expressed in the repaired cells indicated a shift in the VSG switching pathway used. To assess this, we used VSG sequencing (VSG-seq) (59) to examine the number of distinct *VSG* transcripts arising in the population following a VSG switch. The expressed *VSGs* in the VSG^up^ and VSG^upDEX^ cell lines were amplified and sequenced at 0 and 7 days post-DSB induction. *VSG* genes were considered significantly enriched when the log_2_ fold change (FC) of the induced sample compared to the uninduced is >2, with a *P* value of <0.05. We identified 64 *VSG* sequences that were significantly enriched in the VSG^up^ cell line and 66 in the VSG^upDEX^ cell line (Fig. 7A). We next looked at the genomic locations of the significantly enriched genes by mapping them to the T. brucei 427 genome (6) with the minichromosomal *VSGs* added from the VSGnome (7) (Fig. 7B). We found that for the VSG^up^ cell line, 9 *VSGs* were located on the BESs, 20 on the minichromosomes, and 16 on the megabase chromosomes (Fig. 7B and C). For the VSG^upDEX^ cells, the results were strikingly similar, with 9, 20, and 17 *VSGs* located on the BESs, minichromosomes, and megabase chromosomes, respectively (Fig. 7B and C). Therefore, the number and genomic positions of the templates used to repair a VSG^upDEX^ DSB are analogous to that of VSG^up^, indicating that despite the expression and subsequent loss of *VSG-5* following a BES DSB, repair at the BES continues unabated. We then used primers specific to BES1 and *VSG-8* on BES12, which is silent in VSG^up^ and VSG^upDEX^ prior to induction (Fig. S8). No *VSG-8* product was amplified in the uninduced samples, as expected, but products were amplified in both the 196-h induced samples in both VSG^up^ and VSG^upDEX^ (Fig. S8). This suggests that repair of a DSB in BES1 occurs using silent *VSGs* that are gene converted into the active BES. The multiple-banding pattern seen in the induced samples suggests gene conversion events using different lengths of 70-bp repeats for homology. Interestingly, we noted a preference for intrachromosomal repair in both cell lines. *VSG-2* is expressed from chromosome 6, and 8% and 11% of enriched genes aligned to chromosome 6 in the VSG^up^ and VSG^upDEX^ cells, respectively. The preference for intrachromosomal repair may be associated with the proximity of the repair template to the site of the DSB.

10.1128/mbio.03847-21.8 FIG S8 Repair in the VSG^up^ and VSG^upDEX^ cell lines. (Top) Schematic showing BES1 with the SceR site (*) and PAC gene integrated upstream of VSG2. Repair following a DSB by gene conversion using silent *VSG* genes. (Bottom) PCR analysis of the VSG^up^ and VSG^upDEX^ cell lines. Uninduced and 196-hour Tet-induced samples using primers specific to BES1 *pseudo* gene and silent *VSG*, *VSG-8*. White box, genes; P, *VSG pseudo* gene; black box, 70-bp repeats; black circles, telomere; PAC, puromycin resistance cassette; dotted lines, crossover; position of primers indicated by arrows. Schematic created with BioRender.com. Download FIG S8, TIF file, 2.1 MB. Copyright © 2022 McLaughlin et al. 2022 McLaughlin et al. https://creativecommons.org/licenses/by/4.0/ This content is distributed under the terms of the Creative Commons Attribution 4.0 International license.

## DISCUSSION

Here, we report that the expression of a second *VSG* is not tolerated during a VSG switching, with stringent mechanisms to reinforce monoallelic expression in antigenic variation. We disrupted single antigen expression through the integration of an ectopic copy of *VSG-5* approximately 7 kbp upstream of a *de novo* telomere at an rDNA promoter, which drives transcription of *VSG-5* expression while avoiding competition with the native BES promoter (19, 60). Using an rDNA promoter to replace a BES promoter does not disrupt allelic exclusion (61), and the ectopic copy of *VSG-5* is also subject to allelic exclusion (19), with both VSG-2 and VSG-5 present at the cell surface in equal levels. Expression of VSG-2 and VSG-5 was stable, even in the absence of drug selection pressure (Fig. S1B), likely due to the *de novo* telomere seed, which allows for recruitment of the VEX complex and stable transcription of *VSG-5*.

We initially anticipated that expression of *VSG-5* would be coincident with the nucleolus, given the integration of the expression construct at a ribosomal promoter. Our data suggest that the ectopic *VSG-5* locus is expressed from the same position in the nucleus as the active BES and that this leads to an increase in the size of the VEX2 focus at this position. The increase in size is dependent on the expression of two *VSGs*, and we show, using DNA FISH, that *VSG-2* and *VSG-5* transcription is coincident. Indeed, two *VSGs* expressed from distinct BESs colocalize to a single ESB (42). It has previously been reported that ectopic expression from an rDNA locus is achievable but unable to compensate for loss of active *VSG* expression (41); we suggest that the recruitment of the VEX complex by the telomere is necessary for the localization of the ectopic *VSG* locus to the active *VSG* transcription compartment. Previous work by Batram et al. has shown that inducing high levels of expression of a second *VSG* from an rDNA spacer using a T7 RNA polymerase promoter led to initial attenuation of the actively expressed BES *VSG* in a process that is reversed in around 5 days (48). We suggest that in our cell line, the stable expression of *VSG*-2 and *VSG-5* is supported by their proximity to a telomere and their ability to recruit telomeric chromatin components. In the context of this study, we have assessed only one pair of *VSG* genes but believe our findings would be supported using additional pairs of *VSG* genes.

What leads to the transcription-dependent break-induced loss of *VSG-5* is unknown, but one hypothesis is that following a DSB, *VSG-5* transcription increases to compensate for the loss of VSG-2. This could lead to genomic instability through the formation of R-loops (62), which, in T. brucei, have been shown to form in both the active BES (29) and at rDNA loci (30). R-loop-induced genomic instability can result in chromosome rearrangements (63) and could account for the loss of *VSG-5.* Alternatively, increased transcription of *VSG-5* may result in clashes between the transcription and replication machinery, leading to DNA DSBs that are repaired by recombination and resulting in genetic changes (64). In the DEX cells, we observed a significant decrease in ssDNA in the first 6 to 9 h following a DSB. The proximity of the available templates has been shown to influence the frequency of recombination (6), and it is possible that after a DSB in the BES, repair is initiated using the ectopic *VSG-5* gene, which is in close spatial proximity; however, limited homology results in an unsuccessful recombination event which is eventually degraded, as is seen in fission yeast (65). Recombination intermediates could temporarily stall ssDNA formation until the homology search is reestablished. Despite the delay in the timing of DSB repair, the mechanism of repair at the BES continued undisrupted with the preference for repair using the 70-bp repeats maintained and the access to the genomic archive of *VSG* genes unabated.

Two studies have previously reported frequent deletion of an active BES during *VSG* switching. Rudenko et al. used selection markers within the silent BES to activate a transcriptional switch and observed deletion of the originally active BES (66), with up to 100 kbp of telomeric sequence lost and large changes in chromosome size seen. Cross et al. reported a similar finding using a negative selectable marker for BES inactivation, where they found that the majority of switching events were coupled to the deletion of the entire previously active BES, with large changes in chromosome size also seen (67). The mechanism of chromosome truncation is unclear; however, it was proposed that they could arise from either random chromosome breakage and healing as is seen in Plasmodium falciparum (68, 69), interchromosomal recombination, or a failed recombination event that is destroyed by exonucleases (67). Both studies propose that there is a strict requirement for the inactivation of the previously active *VSG* prior to activation of a new one, either by a transcriptional switch or deletion of the previously active BES (66, 67). The mechanisms by which expressed *VSG* genes are counted to ensure monoallelic expression are not fully understood; however, it has been proposed that sequences in the *VSG* gene and downstream sequences, including the telomeric TTAGGG repeats, which share a high degree of homology with other BESs, could act as repressive elements in a VEX-dependent manner (19). The ectopic *VSG-5* expression construct used in this study contains the native *VSG-2* 3′ UTR and associated downstream sequences and a *de novo* telomere which facilitates recruitment of the VEX complex, allowing it to escape *VSG* silencing (19). Given the close proximity of the ectopically expressed *VSG-5* and native *VSG-2* alleles in the nucleus, an I-*Sce*I-induced DSB may cause a transient release of the VEX complex from both, but only recruited back to the repaired BES expressing a new *VSG* gene. This would leave the ectopic locus depleted of the VEX complex, impairing expression.

Here, we report that monoallelic expression is rapidly restored during a VSG switching event. We propose that this occurs in order to prevent multiple VSG switching events from occurring at once, preserving monoallelic expression. It is of note that a number of studies into VSG switching have been unable to distinguish between BES GC, in which an entire BES is duplicated and replaces the previously active one, and an *in situ* switch coupled to deletion of the originally active BES (35, 39, 70–72). The resulting structure of the active BES is identical in both cases, and the loss of the previously active BES will only be identified by examining chromosome truncations. Therefore, it is possible that deletion of the previously active BES occurs frequently during a VSG switch; however, these events have so far gone largely undetected.

T. brucei evasion of the mammalian host is dependent on both the monoallelic expression of a single surface antigen in order to avoid clearance by the host immune system (18) and the periodic switching of the single expressed *VSG* species. Here, we demonstrate that monoallelic expression is maintained at an unexpected level during antigenic variation, which has important implications for our understanding of how the parasite evades immune detection.

## MATERIALS AND METHODS

### T. brucei cell growth and manipulation.

T. brucei Lister 427 cell lines were grown in HMI-11 medium at 37.4°C (73) with 5% CO_2_, and the density of cell cultures was measured using a hemocytometer. Wild-type (WT) cell lines used in this study were T. brucei Lister 427 MITat 1.2 clone 221a (VSG-2 WT or VSG221 WT) and MITat 1.5 (VSG-5 WT, or VSG118 WT). Transformation of cell lines was carried out by centrifuging 2.5 × 10^7^ cells at 1,000 × *g* for 10 min at room temperature. The cell pellet was resuspended with 10 μg linearized DNA in 100 μL warm cytomix solution (74), placed in a cuvette (0.2-cm gap), and transformed using a Nucleofector (Lonza) (X-001 function) (75). Transfected cells were recovered in 36 mL of warm HMI-11 at 37°C for 4 to 6 h, after which cells were plated out in 48-well plates with the required drug selection. G418 selection was carried out at 2 μg/mL, puromycin at 2 μg/mL, blasticidin at 10 μg/mL, and tetracycline (Tet) at 1 μg/mL. Puromycin, phleomycin, hygromycin, blasticidin, and G418 selection was maintained at 1 μg/mL. The VSG^up^ cell line has been described previously (26). The VSG^upDEX^ cell line was generated by transfecting the VSG^up^ cell line with the p^5^NTMF reporter construct (19) and clones with detectable *VSG-5* expression selected. The VSG^upDEX_OFF^ cell line was generated in the same way; however, cells that do not express *VSG-5* were selected.

To measure cumulative cell growth, a hemocytometer was used, and cells were diluted to 1 × 10^5^ cells/mL every 24 h. DSB induction was carried out with 1 μg/mL tetracycline. A pair of clones was analyzed for VSG^upDEX^. Generation of subclones was carried out by clonogenic assays in which VSG^up^ and VSG^upDEX^ cells were plated out in 96-well plates. For the uninduced (−Tet) plates, 32 cells were seeded per 96-well plate, and for the induced (+Tet), 480 cells were seeded per plate and induction carried out using 1 μg/mL tetracycline for 4 to 7 days. Cells grown under induced conditions had puromycin and G418 drug selection removed. DSB induction was confirmed in all induced subclones using a puromycin-sensitivity assay in which induced subclones were grown in the absence and presence of 2 μg/mL puromycin, and only those clones that did not grow in the presence of puromycin were considered sensitive.

### Plasmids.

The p^5^NTMF plasmid (19) was linearized using SmaI. Epitope tagging of VEX2 at the native locus was achieved using the VEX2^6myc^ plasmid (20), digested using SphI.

### Immunofluorescence analysis.

Immunofluorescence was carried out according to standard protocols. In brief, cells were fixed in 1% (vol/vol) formaldehyde and incubated on ice for 30 min. Fixed cells were centrifuged for 1 min at 6,000 rpm and washed with 1 mL ice-cold phosphate-buffered saline (PBS) twice. Cells were settled onto poly-l-lysine-treated slides for up to 30 min and washed 3× for 5 min in PBS. Blocking was carried out for 15 min in 50% fetal bovine serum (FBS) in PBS, and all antibody dilutions were in 3% FBS. For visualization of internal antigens, antigen retrieval was carried out prior to blocking by incubation at 95°C for 1 min in antigen retrieval buffer (100 mM Tris, 5% urea, pH 9.5) as described in reference 19. Primary antisera used were rat α-VSG-2 (1: 50,000), rabbit α- VSG-5 (1: 50,000), rabbit α-myc (Cell Signaling; catalog no. 71D10) (1:200), and mouse α-L1C6 (1:100) (53), a gift from the Bastin laboratory. Secondary antisera used were goat α-rat rhodamine conjugated (1:100), goat α-mouse fluorescein isothiocyanate (FITC) conjugated (1:100), both Invitrogen, and goat α-rabbit Alexa Fluor 555 and goat α-mouse Alexa Fluor 488, both used at 1:2,000 dilution and provided by Thermo Fisher. Cells were mounted in Vectashield (Vector Laboratories) containing 4,6-diamidino-2-phenylindole (DAPI). DAPI-stained nuclei and kinetoplast were used as cytological markers to determine the cell cycle stage (76). Cells with one nucleus and one kinetoplast are G_1_, one nucleus and one elongated kinetoplast (IN:1eK) are S phase, one nucleus and two kinetoplasts (1N:2K) are G_2_/M, and cells with two nuclei and two kinetoplasts are postmitosis. Images were acquired using a Zeiss Axio Imager Z2 epifluorescence microscope combined with an Axiocam 506 monocamera. Images were processed using ImageJ, version 2.1.0 (77). Statistical analysis was carried out in GraphPad Prism, version 9.

### Protein analysis.

Protein analysis was carried out using standard procedures (78). Primary antisera used were rabbit α-VSG-2 and α-VSG-5, both at 1:50,000; and rabbit α-histone H3 at 1:100,000, rabbit α-NPT at 1:8,000 (Fitzgerald), and mouse α-EIF1α at 1:20,000 (Millipore). Secondary antibodies used were horseradish peroxidase-coupled goat α-rabbit and goat α-mouse (Bio-Rad), used at 1:1,000. Protein blots were developed using an enhanced chemiluminescence kit (Amersham).

### Flow cytometry.

Flow cytometry experiments were performed as described in reference 19. Approximately 1 × 10^7^ cells were collected for analysis for each sample. VSGs were detected using mouse α-VSG-5 (1:10,000) and rat α-VSG-2 (1:10,000) primary antibodies. Secondary antibodies used were goat α-rabbit Alexa Fluor 488 (Thermo Fisher) and goat α-rat Alexa Fluor 647 (Thermo Fisher), both at 1:2,000 dilution. Samples were analyzed on a MoFlo Astrios EQ cell sorter, and data were analyzed using FlowJo software.

### DNA analysis.

Primers were used to amplify *VSG-2*, *ESAG1*, and *Pseudo* genes as described in reference 26. Primers used to amplify *NPT* were Neo.2 F (GATGGATTGCACGCAGGTTCTC) and Neo.2 R (CCTTGAGCCTGGCGAACAG), and to amplify the start of *VSG-5* were rP2 F (AGATTAAGCAGTAAAAGTAGCGC) and VSG-5 R (CTTTTGTAGTTTTAGGCCGTGATTG).

### DNA FISH.

DNA probes were generated by PCR using standard conditions with Phusion high-fidelity DNA polymerase (Thermo Scientific) and labeled using FISH Tag DNA multicolor kit (Invitrogen). For the *Pseudo* gene probe, a 1,328-bp fragment was amplified with PSEUDO_FISHF (AACAGCGCCGAATTTAATGCAAT) and PSEUDO_FISHR (GTTTCGCCTTCCCATTTGCAT) primers and labeled with Alexa Fluor 488 dye (Invitrogen). For the NTP probe, a 1,200-bp fragment was amplified from the pTMF-8U2 plasmid using NTP_FW CTAACAGGCACGGAAGCCTA and NTP_RV CTCCAAAGCAAACATGCAGA primers and labeled with Alexa Fluor 555 dye. Synthesis and labeling of the amine-modified DNA were done following the manufacturer’s conditions (Invitrogen) using 1 μg of purified amplification product. For hybridization, each DNA probe was used at a final concentration of 10 ng/μL in hybridization buffer (50% formamide, 10% dextran sulfate, 2× SSC [1× SSC is 0.15 M NaCl plus 0.015 M sodium citrate]) containing herring sperm DNA (10 μg/mL; Sigma) and yeast tRNA (10 μg/mL; Invitrogen).

FISH protocol was adapted from Budzak et al. (42). Cells were fixed directly in medium with ice-cold paraformaldehyde at a final concentration of 4% for 10 min in ice. Cells were washed three times in cold 1× PBS buffer and settled within previously adhered Gene Frames (Thermo Scientific) on Polysine microscopy slides for 30 min. Cell permeabilization was made with 0.1% Nonidet P-40 for 10 min and washed three times in 1× PBS. RNase treatment was carried out with 50 μg/mL RNAse A (Thermo Scientific) for 1 h at 37°C. We added 25 μL of probe mix to the slide before placing the Gene Frames coverslip; denaturation was carried out at 82°C for 5 min, followed by overnight incubation at 37°C in a wet chamber. After hybridization, slides were washed in DNA wash buffer (50% formamide, 2× SSC) for 30 min at 37°C, followed by washes in 1× SSC, 2× SSC, and 4× SSC at 50°C for 10 min each. Slides were dipped in water and air-dried, followed by counterstaining with 1 μg/mL Hoechst for 10 min in the dark. Samples were rinsed in 1× PBS and mounted in SlowFade Gold antifade (Invitrogen). Images were acquired using a Zeiss Axio Imager Z2 epifluorescence microscope combined with an Axiocam 506 monocamera. Images were processed using ImageJ, version 2.1.0 (77). Statistical analysis was carried out in GraphPad Prism, version 9.

### Whole-genome sequencing.

Two uninduced and four 7-day DSB-induced VSG^upDEX^ subclones were harvested at 5 × 10^7^ cells and DNA extracted using a Qiagen DNeasy blood and tissue kit according to the standard protocol. DNA was sequenced on a BGISEQ-500 platform in paired-end mode at the Beijing Genomics Institute (BGI). The number of reads per library was as follows: 10.92 million for VSG^upDEX^ uninduced 1, 12.05 million for VSG^upDEX^ uninduced 2, 10.91 million for VSG^upDEX^ induced 1, 11.63 million for VSG^upDEX^ induced 2, 12.86 million for VSG^upDEX^ induced 3, and 12.77 million for VSG^upDEX^ induced 4. Sequences were aligned to the T. brucei Lister 427 genome (6), with the minichromosomal *VSGs* (5) and p^5^NTMF sequences added, using Bowtie2 (79) with “–very-sensitive” parameters and BAM files generated using SAMtools (80). The percentages of reads successfully aligning to the genome were as follows: 96.70% for VSG^upDEX^ uninduced 1, 99.03% for VSG^upDEX^ uninduced 2, 98.19% for VSG^upDEX^ induced 1, 99.03% for VSG^upDEX^ induced 2, 98.59% for VSG^upDEX^ induced 3, and 98.94% for VSG^upDEX^ induced 4. The genome was visualized using Artemis genome browser (81), and reads were filtered for uniqueness using a mapping quality (MAPQ) cutoff of 1.

### Quantitative resection assay.

Quantification of ssDNA by qPCR was carried out using an adapted version of reference 57. In brief, DNA was harvested at 0, 3, 6, 9, and 12 h following growth in 1 μg/mL tetracycline. Five hundred nanograms of extracted DNA were digested with either HindIII or mock digested (no enzyme) overnight at 37°C. For the quantitative PCR (qPCR), primers used were VSG21b forward (AGGCCAAGAAAGCGCTAACA) and VSG21b reverse (CCACTGGCTGCTCGGATATG) (55). Luna Universal qPCR master mix (New England Biolabs) was used with 600 pM primer mix and 5 ng DNA per reaction. The PCR cycling conditions were 95°C for 3 min and then 40 cycles of 95°C for 10 s and 55°C for 30 s on a thermal cycler. The quantification cycle (Δ*C_q_*) was calculated by subtracting the average *C_q_* of the mock digest from the digested *C_q_*. The percentage of ssDNA was calculated using the following formula: % resection = 100/[(1 + 2^Δcq^)/2], assuming 100% efficiency of the I-*Sce*I meganuclease. Three technical replicates were carried out for each experiment. Statistical analysis was carried out in GraphPad Prism, version 9.

### *VSG* sequencing.

For both the VSG^up^ and VSG^upDEX^ cell lines, 5 × 10^7^ cells were harvested from uninduced and 7-day DSB-induced cells, and RNA was extracted using an RNeasy RNA extraction kit (Qiagen). For the VSG^up^ cell line, experiments were carried out in duplicate, and VSG^upDEX^ experiments were carried out in triplicate. First-strand synthesis was performed using 500 ng of RNA, SuperScript IV reverse transcriptase (Thermo Fisher), and 200 nM “All-*VSG* 3′-UTR” primer (GTGTTAAAATATATC) (59) that binds specifically to the conserved 14-mer in the *VSG* 3′ UTR. The product was cleaned up using AmPureXP beads (Beckman Coulter). To specifically amplify all of the *VSG*s expressed in a population, conserved sequences in the 5′ spliced leader and 3′ UTR of every *VSG* mRNA were used (59). *VSG* cDNA was amplified by PCR using 1 μg of cDNA, 0.2 mM deoxynucleoside triphosphates (dNTPs) (NEB), 1× PCR buffer (NEB), Phusion DNA polymerase (NEB), 200 nM spliced leader (SL) forward primer (ACAGTTTCTGTACTATATTG), and 200 nM SP6-14mer reverse primer (GATTTAGGTGACACTATAGTGTTAAAATATATC) (59). The PCR conditions were 5 cycles of 94°C for 30 s, 50°C for 30 s, and 72°C for 2 min, followed by 18 cycles of: 94°C for 30 s, 55°C for 30 s, and 72°C for 2 min carried out on a thermal cycler machine. The *VSG* PCR products were then cleaned up using AmpPureXP beads (Beckman Coulter) according to the manufacturer’s protocols. Sequencing was carried out using a minimum of 4 μg product per sample at Beijing Genomics Institute (BGI) using the BGISEQ-500 platform in paired-end mode. The number of million reads per library was 9.21 for VSG^up^ uninduced replicate 1, 9.29 for VSG^up^ uninduced replicate 2, 8.01 for VSG^up^ induced replicate 1, 7.98 for VSG^up^ induced replicate 2, 9.50 for VSG^upDEX^ uninduced replicate 1, 9.63 for VSG^upDEX^ uninduced replicate 2, 8.42 for VSG^upDEX^ uninduced replicate 3, 8.02 for VSG^upDEX^ induced replicate 1, 8.02 for VSG^upDEX^ induced replicate 2, and 8.06 for VSG^upDEX^ induced replicate 3. Reads were aligned to the T. brucei Lister 427 genome (6) with the minichromosomal *VSGs* added from the VSGnome (7) using Bowtie2 (80) with “–very-sensitive” parameters and BAM files generated using SAMtools (80). The overall alignment was 95.21% for VSG^up^ uninduced replicate 1, 95.24% for VSG^up^ uninduced replicate 2, 97.51% for VSG^up^ induced replicate 1, 97.08% for VSG^up^ induced replicate 2, 97.08% for VSG^upDEX^ uninduced replicate 1, 97.83% for VSG^upDEX^ uninduced replicate 2, 97.81% for VSG^upDEX^ uninduced replicate 3, 98.41% for VSG^upDEX^ induced replicate 1, 98.52% for VSG^upDEX^ induced replicate 2, and 99.08% for VSG^upDEX^ for VSG^upDEX^ induced replicate 3. Reads aligning to each transcript were acquired using featureCounts (82), and EdgeR (83) was used to perform differential expression analysis on all genes. The R script used to generate the volcano and genome plots and perform differential genome analysis was adapted from reference 55.

### Data availability.

Whole-genome sequencing data have been deposited onto the ENA under study accession number PRJEB43910 and unique study name ena-STUDY-INSTITUT PASTEUR-24-03-2021-19:28:45:086-149. Transcriptomic data have been deposited onto the ENA under study accession number PRJEB49263.
