# Supplementary figures and images for: Transcription Dependent Loss of an Ectopically Expressed Variant Surface Glycoprotein during Antigenic Variation in Trypanosoma brucei
Source: mBio. 2022 Mar 1;13(2):e03847-21. doi: 10.1128/mbio.03847-21 (PMC8941856; doi:10.1128/mbio.03847-21)

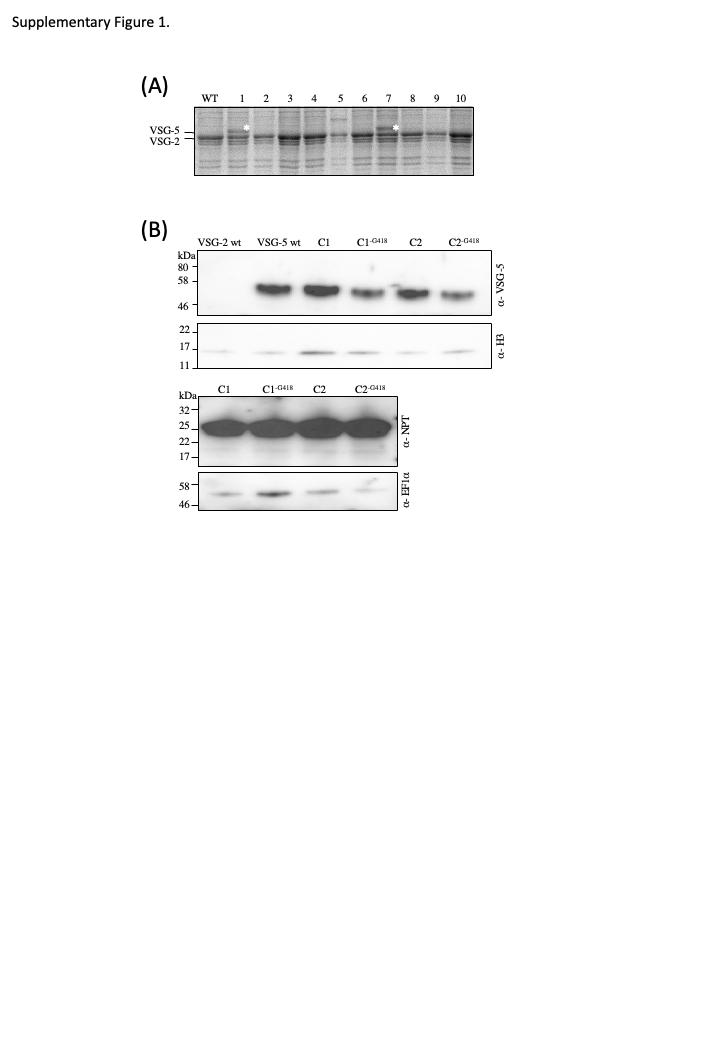

Supplement: FIG S1 [file mbio.03847-21-sf001.tif]

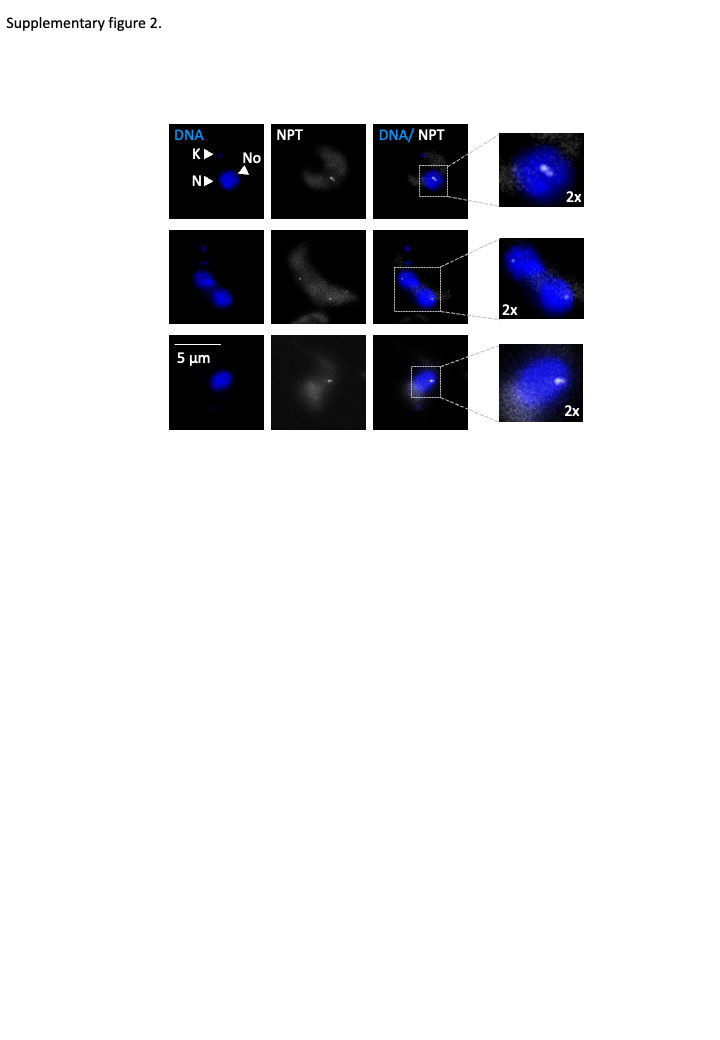

Supplement: FIG S2 [file mbio.03847-21-sf002.tif]

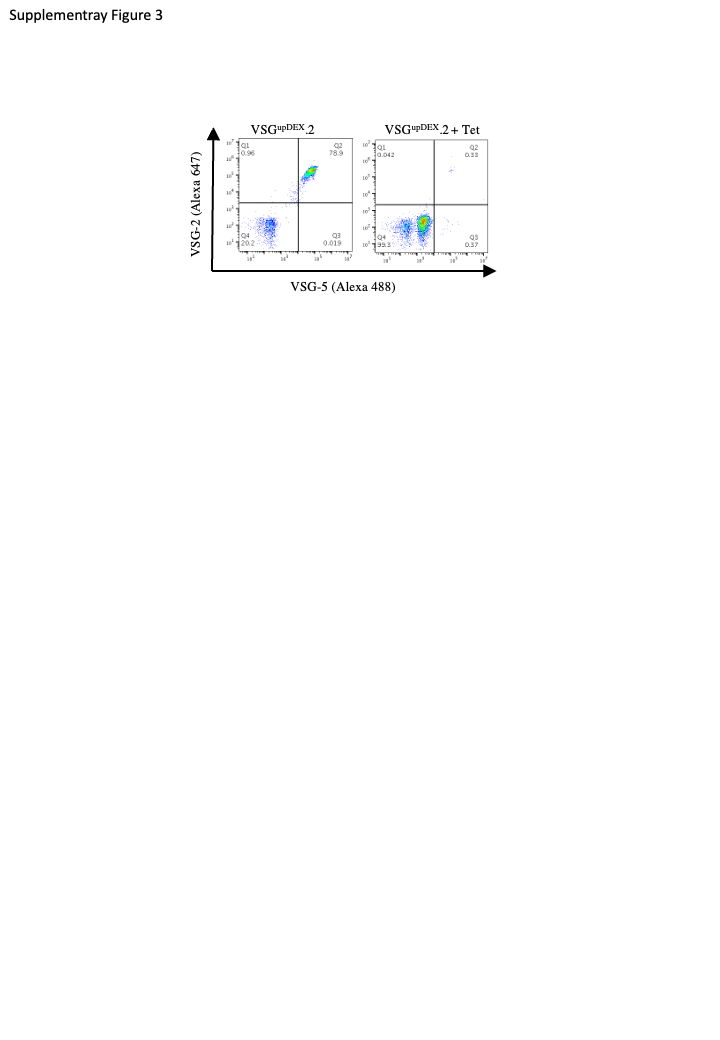

Supplement: FIG S3 [file mbio.03847-21-sf003.tif]

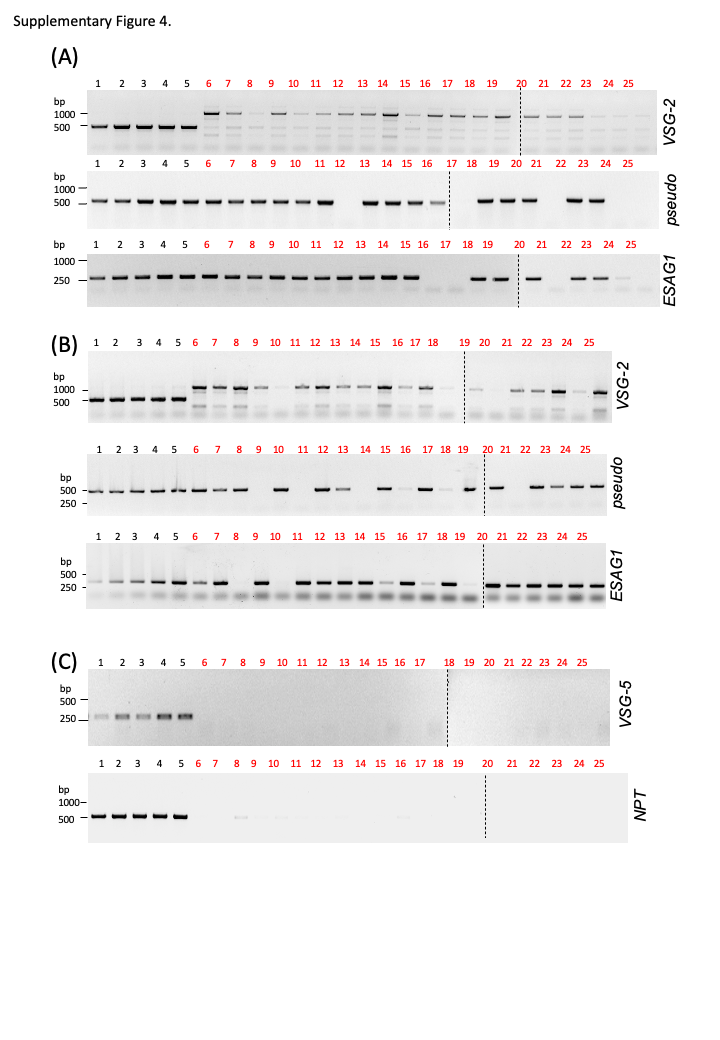

Supplement: FIG S4 [file mbio.03847-21-sf004.tif]

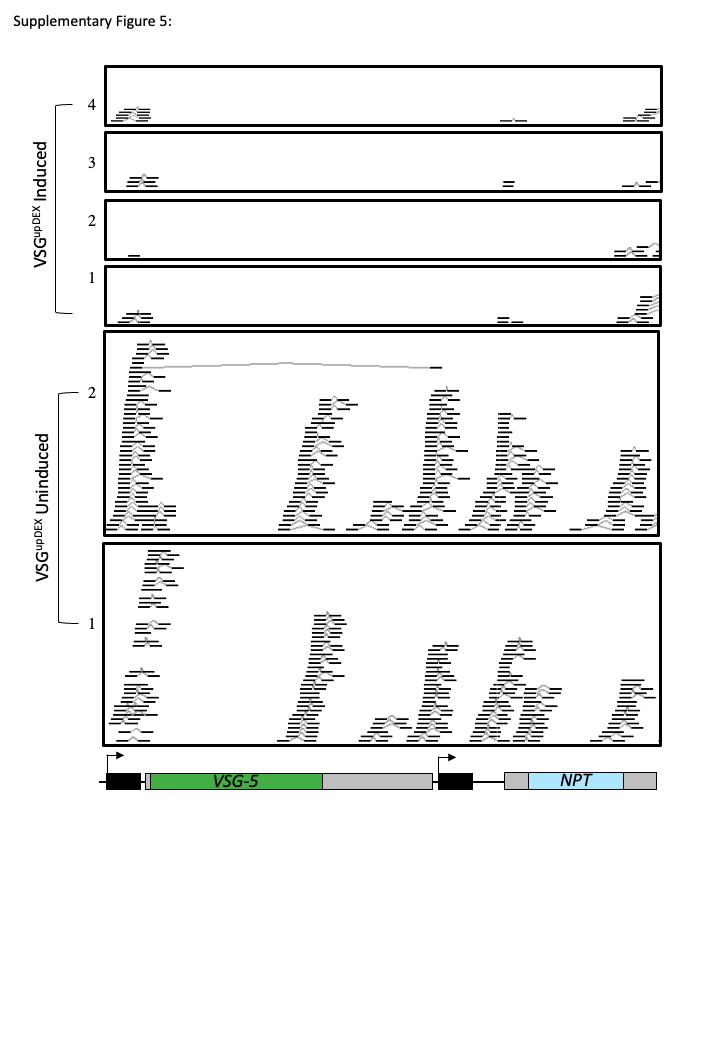

Supplement: FIG S5 [file mbio.03847-21-sf005.tif]

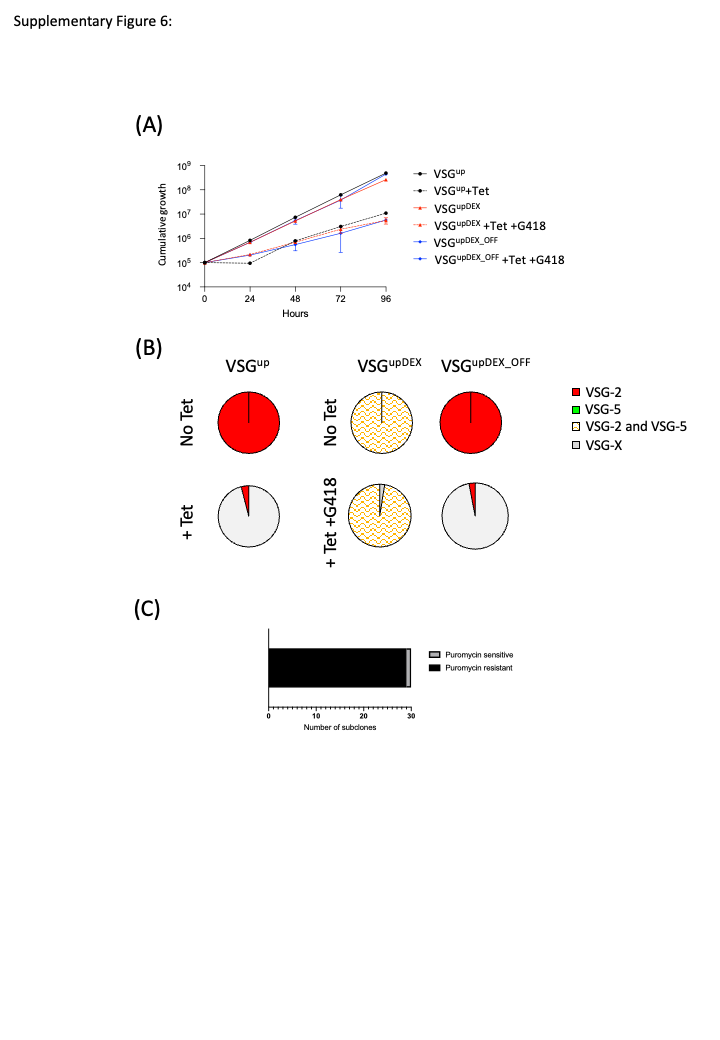

Supplement: FIG S6 [file mbio.03847-21-sf006.tif]

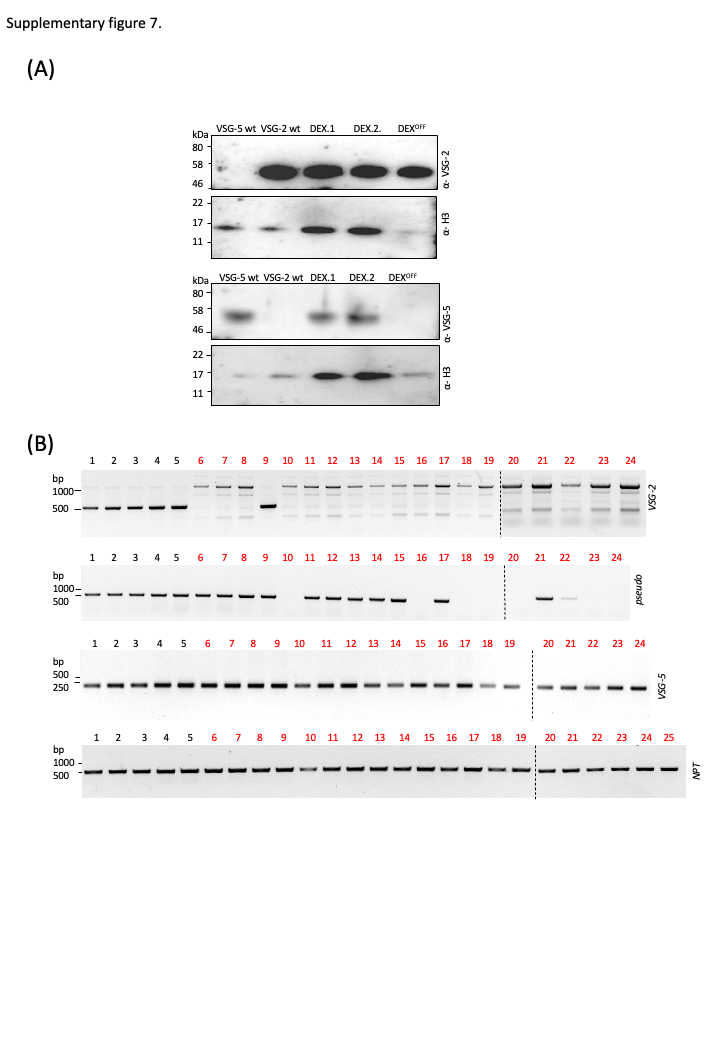

Supplement: FIG S7 [file mbio.03847-21-sf007.tif]

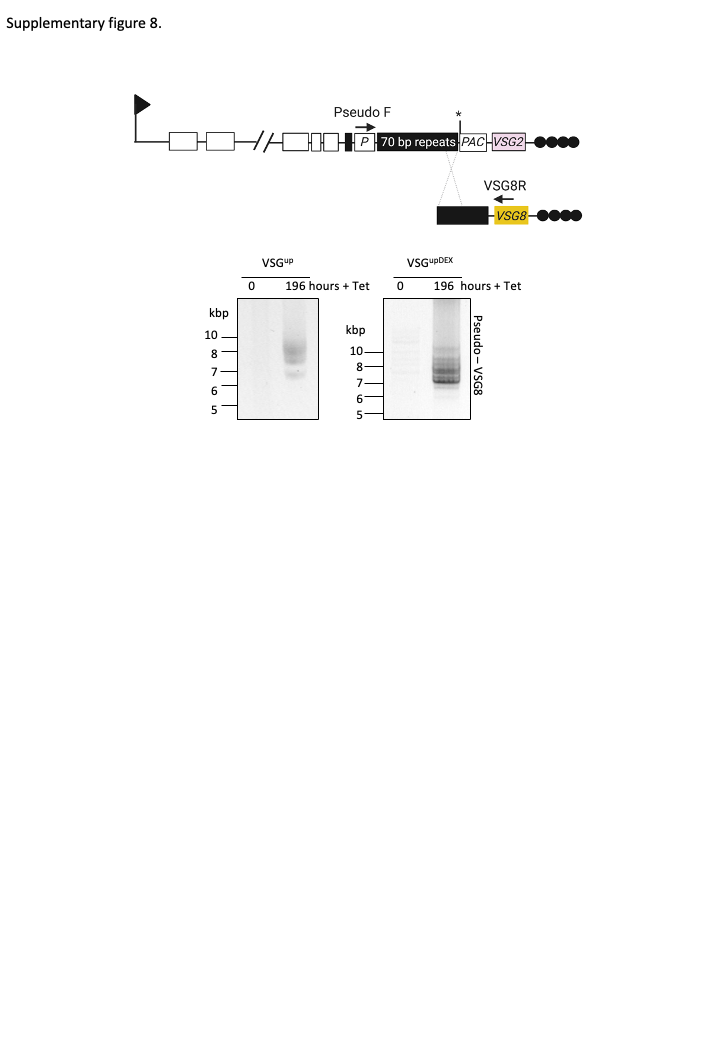

Supplement: FIG S8 [file mbio.03847-21-sf008.tif]
